# Black Phosphorus-New Nanostructured Material for Humidity Sensors: Achievements and Limitations

**DOI:** 10.3390/s19051010

**Published:** 2019-02-27

**Authors:** Ghenadii Korotcenkov

**Affiliations:** Laboratory of Physics and Engineering of Nanomaterials, Department of Physics and Engineering, Moldova State University, MD-2009 Chisinau, Moldova; ghkoro@yahoo.com

**Keywords:** phosphorene, parameters, synthesis, fabrication, characteriszation, advantages, limitations, tunability, stability

## Abstract

The prospects of using nanostructured black phosphorus for the development of humidity sensors are considered. It was shown that black phosphorus has a set of parameters that distinguish it from other two-dimensional (2D) materials such as graphene, silicone, and dichalcogenides. At the same time, an analysis of shortcomings, limiting the use of black phosphorus as a humidity sensitive material in devices aimed for market of humidity sensors, was also conducted.

## 1. Introduction

Due to the unique properties of water, humidity strongly affects materials and living organisms, including humans [1,2]. The amount of water vapor in the air can affect the human comfort and the efficiency and the safety of many manufacturing processes. In particular, humidity affects drying of paint, paper, matches, fur and leather, packaging and storage of tea, cereal, milk, and bakery items, and manufacturing of various products such as food, plywood, gum, abrasives, pharmaceutical powder and tablets, ceramics, and printing materials [2,3]. Moreover, the industries discussed above are only a few of the industries where it is necessary to control humidity. In agriculture, using humidity sensors, one can monitor the humidity of the air in greenhouses, the presence of a sufficient amount of moisture in the soil, organize the plantation protection (dew prevention), and so on [4]. As for medicine, respiratory equipment, sterilizers, incubators, pharmaceutical processing, and the storage of biological products also require constant moisture control. Humidity measurements at the Earth’s surface as well as in the stratosphere and troposphere are also required for climate studies, for meteorological analysis and forecasting, and for many special applications in hydrology, aeronautical services, and environmental studies, since water vapor is the key agent in both weather and climate [2,5]. This means that determining and measuring humidity is of great importance. 

In the past decade, much has been done to develop new methods for measuring humidity, to improve and optimize the manufacturing technology of already developed humidity sensors, and also to develop various measuring systems with increased efficiency [6,7,8,9,10,11,12,13]. However, the process of developing new humidity sensors and improving older types of devices used for humidity measurement is still ongoing. New technologies and the toughening of ecological standards require more sensitive instruments with faster response times, better selectivity, and improved stability. This means that we need new humidity-sensitive materials that would solve these problems [13].

## 2. Black Phosphorus

Phosphorus is quite common in the Earth’s crust. Its content is 0.08–0.09% of the total mass of the Earth’s crust. Phosphorus has several allotropic modifications. All these modifications are different in their properties. The most common are white phosphorus, yellow phosphorus, red phosphorus, and black phosphorus. White phosphorus is most active. It self-ignites easily, glows in the dark and is highly poisonous. Red phosphorus (RP) is less chemically active and less toxic. Red phosphorous oxidizes slowly in air and self-ignites only at elevated temperatures (*T* > 240 °C). Bulk black phosphorus (BP) is the least active of all modifications. It does not oxidize in air. Moreover, black phosphorus is a semiconductor material with unusual electronic and optical properties [14,15]. However, despite this, black phosphorus, due to structural instability and toxicity, has so far had very limited use. The main consumer of phosphorus was agriculture (phosphate fertilizers), and the production of synthetic detergents, phosphate glasses, explosives and matches. Only recent studies have shown that new applications can open up for black phosphorus. These prospects are primarily associated with the discovery of phosphorene, a new two-dimensional (2D) material [16,17,18,19,20,21,22,23,24,25,26,27,28,29,30,31,32]. The main distinguishing feature of 2D materials is the ability to form so-called nanosheets, which can be micrometers wide, but less than a nanometer thick. Such materials with extraordinary great values of total surface area (>1000 mm^2^/g) can have dramatically enhanced surface activity, necessary for applications in various fields.

At present, a sufficiently large amount of 2D materials is known. But the most popular and studied are two-dimensional materials such as graphene, silicine, transition metal dichalcogenides (TMDCs), and hexagonal boron nitride (h-BN). These materials have unique parameters. But at the same time, they are characterized by disadvantages. In the case of graphene, an allotrope form of carbon consisting of a single layer of carbon atoms arranged in a hexagonal lattice, this is the absence of the band gap, which reduces graphene applicability in semiconducting and optoelectronic devices. Silicene, the two-dimensional layer form of silicon, has insufficient resistance to environmental influences. Silicon when exposed to air is easily oxidized. Metal dichalcogenides have direct band gap and strong light absorption that can be utilized in the optoelectronic devices. However, the Mo- and W-based TMDCs, most promising 2D materials, are relatively wide-gap materials (see Table 1). Furthermore, TMDCs have relatively low mobility of charge carriers when compared to graphene and phosphorene. These drawbacks of TMDCs hinder both the improvement of the performance of electronic devices, and optoelectronic and photonic applications, which require a different band gap range. As for hexagonal boron nitride (h-BN), it has too wide band gap (E_g_ > 5 eV). In addition, h-BN is an insulating material. With this regard experiment and simulations have shown that 2D black phosphorus (2D BP) does not have many of the shortcomings of the two-dimensional materials mentioned above.

### 2.1. Black Phosphorus and Its Crystal and Electronic Structures

**Figure 1:**
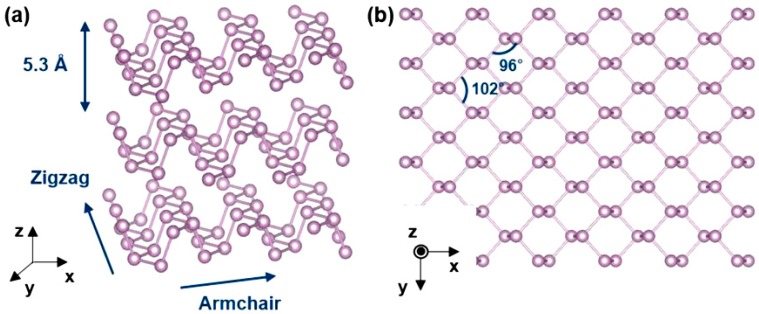
(**a**,**b**) Schematic diagram of the crystal structure of black phosphorus. The system is relaxed using density functional theory calculation. Reproduced with permission from Ref. [38]. Copyright American Chemical Society, 2009.

Black phosphorus forms a layered structure with single element, phosphorus, just like graphene [34,35]. The top view of the hexagonal structure of black phosphorus with bond angles 102.09° and 96.34° is shown in Figure 1b. However, the BP structure has several differences when compared with other layered materials consisting of group IV elements. In its layered structure, phosphorus atoms have five valence electrons which have configuration of 3s^2^3p^3^ valence shell. To build the bond with other atoms, each phosphorus atom goes through hybridization and forms sp^3^ hybridized orbitals. These orbitals make covalent bonding with adjacent four phosphorus atoms and this constructs a puckered structure (see Figure 1a). Because of its puckered structure, a single layer of black phosphorus consists of two atomic layers and includes two kinds of interatomic bonds. A shorter bond with a length of 0.2224 nm connects phosphorus in the same layer, and a longer bond with a length of 0.2244 nm connects phosphorus at the top and bottom of this layer [34,36]. Figure 1a shows that black phosphorous has layered structure with space of 0.53 nm. An important feature of this structure is weak van der Waals interaction between the layers and strong covalent intra-layer interaction [37]. This means that BP has two fundamentally different types of bonds. In addition, in BP structure there are also two distinctive directions, zigzag, and armchair. The presence of these two different directions leads to strong in-plane anisotropy in the black phosphorus properties such as effective masses, phonon dispersions, thermal transport, and magnetic properties. 

The comparison of the electriophysical properties of 2D BP and other most studied 2D materials is given in Table 1. It can be seen that the bulk BP, which is a p-type semiconductor with a direct band gap (E_g_ ~0.34 eV), is characterized by good electrical conductivity (≈10^2^ S/m) and the high mobility of electrons and holes, 220 and 350 cm^2^/V·s, respectively [36,39]. Studies have also shown that 2D BP has three crystalline phases: orthorhombic, rhombohedral, and simple cubic phases [40]. Of these phases, the orthorhombic phase has semiconductor properties. This phase is stable under environmental conditions, but at elevated pressure (P ~5.5 GPa) it can transform into a semimetallic rhombohedral phase [41]. At a much higher pressure (P > 10 GPa), the semimetallic rhombohedral structure transforms into a metallic cubic phase [42]. The changes occurring are due to internal distortion: since the Van der Waals interlayer bond is relatively weak, the distance between the individual layers decreases faster with increasing pressure than the intralayer atomic separations [43].

**Figure 2:**
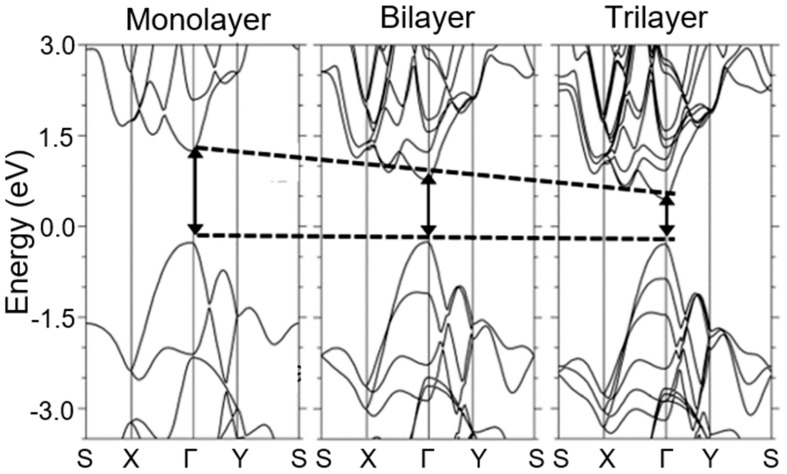
Electronic structures of monolayer, bilayer and trilayer black phosphorus calculated using first principles. Direct band gap of each layer is presented with black arrow at gamma position. The energy is scaled with respect to the Fermi energy. Reproduced from Ref. [53]. Published by IOP Publishing LTD as open access, 2014.

Regarding 2D BP with a layer thickness close to monolayer (also termed “phosphorene”), then the description of the electronic structure and the electronic properties of this material can be found in Refs. [34,44,45,46,47,48,49,50]. The most important results obtained in the study of such structures are shown in Figure 2, which depicts the band structures of monolayer, two-layer and three-layer black phosphorus. The above band structures were built based on the results of a theoretical calculation based on the density functional theory (DFT) with HSE06 hybrid functional. Simulations have shown that compared with other two-dimensional materials, the electronic structure of 2D black phosphorus has some distinct properties. 2D black phosphorus has a direct band gap at the Γ point of the Brillouin zone, regardless of its number of layers. At that the shapes of the band in single layer, bilayer and three-layered BP are similar to each other, and they have the same translational structural symmetry and bond interactions [51,52]. For comparison, transition metal dichalcogenides (TMDCs) have direct band gaps at the K point only for the single layers, while multilayer 2D TMDCs usually have indirect band gaps. 

**Figure 3:**
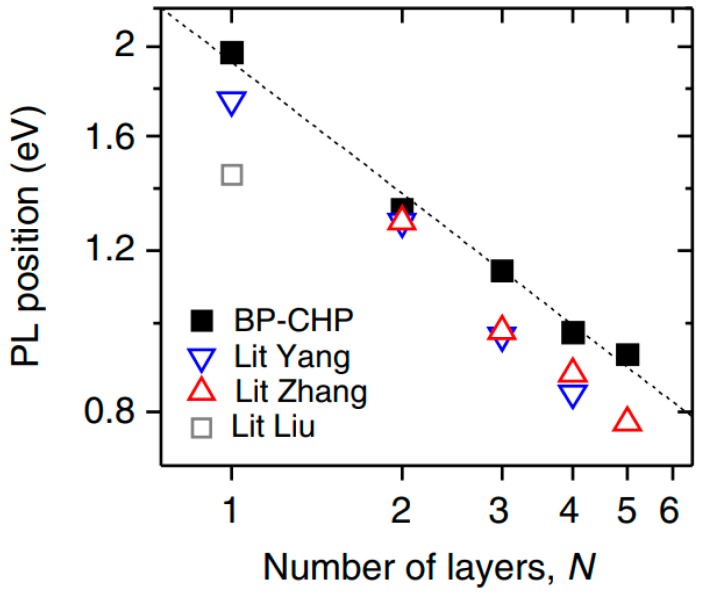
Photoluminescence of black phosphorous nanosheets. Position of PL lines plotted versus layer number: ■—BP nanosheets were exfoliated by sonication in solvent of N-cyclohexyl-2-pyrrolidone (CHP); ∆, ▼, □—data for mechanically cleaved BP nanosheets taken from Refs. [54,57,58]. Reproduced from Ref. [59]. Published by Macmillan Publishers Limited as open access, 2015.

It is also seen in the Figure 3 that the band gap of 2D BP depends on the number of layers. If the monolayer BP has a direct band gap of ~2.0 eV, then in the three-layer structure the band gap is reduced to ~1.0 eV [17]. It is important to note that the results of theoretical modeling were confirmed experimentally. In particular, Liu et al. [54] investigated the photoluminescence of the phosphorene in the visible spectral region and showed that the band gap in phosphorene (1.45 eV) was really significantly larger than in bulk BP (~0.3 eV) [39]. Other data related to the study of the photoluminescent properties of 2D BP with different numbers of layers are shown in Figure 3. Thus, theoretical and experimental studies indicate that depending on the BP thickness, black phosphorus may have bandgap values ranging from 0.3 eV in the bulk material to 2.0 eV in a single layer material, phosphorene [45]. It indicates that the tunable bandgap of BP closes the gap between 0 eV (graphene) and 1.0–2.0 eV (TMDCs) [55,56]. In other words, the energy range of BP band gap covers the visible, near-IR (near-infrared) and mid-IR parts of the electromagnetic wave spectrum. 

Another important feature of 2D black phosphorus is its electro-physical properties. Theoretical simulations based on first-principle calculations showed that effective masses of both electron and hole decrease, when the number of black phosphorus layer increases [60]. In this case, the effective hole mass is always less than the effective mass of electrons. This means that the conductivity of the holes surpasses electron conductivity, making the holes as the major charge carriers in black phosphorus. In other words, in a normal state, 2D BP is a *p*-type semiconductor, which would seem to limit the possibility of forming various BP-based structures that require *n*-type conductivity materials. However, it was established that this problem can be solved and *n*-type black phosphorus can be achieved by doping. In particular, Xu et al. [50] have shown that tellurium can be used as such a dopant. Xu et al. [50] have found that with partial replacement of phosphorus atoms by tellurium atoms doped black phosphorus had *n*-type conductivity. The same effect was also observed when BP was doped with copper [61]. Yu et al. [62] established that chemical doping with benzyl viologen can also be used as an effective electron doping strategy to obtain BP with *n*-type conductivity. One should note that the ability to realize both *n*-type and *p*-type conductivity is a unique property of 2D BP, including phosphorene [63]. For comparison, graphene is semimetal, and most other 2D semiconductors, known till now, are materials with *n*-type conductivity. If you compare the field-effect mobility of charge carriers in 2D materials, it turns out that the mobility of charge carriers in phosphorene (almost 1000 cm^2^/V·s along armchair direction) is lower than in graphene (10,000–15,000 cm^2^/V·s) [64]. However, it is much higher than in 2D TMDCs and even higher than in typical silicon-based devices (about 500 cm^2^/V·s).

Simulations have shown that phosphorene is also a new-emerging two-dimensional material with fascinating thermal properties. It was established that electronic, phononic, and thermoelectric transport properties of single layer BP allow to hope for the achievement of extremely high values of thermoelectric figure of merit, ZT (the criterion for commercial deployment); ZT is proportional to the square of Seebeck coefficient, and the ratio of an electrical conductance of thermoelectric material to its thermal conductance. With a regular decrease of the lattice thermal conductivity in phosphorene compared with bulk BP, ZT can reach more than 2.5 for elevated temperatures (500 K) [65,66]. As is known, for practical applications figure of merit should be more than 1.0, and one of the approaches to increase ZT is to increase the operating temperature. For comparison, due to extremely high thermal conductivity (~2000 W/m·K) at room temperature [33], graphene is not considered as a potential candidate for thermoelectric applications.

The listed set of properties of BP means that 2D black phosphorous is a promising material for the development of electronic and photoelectric sensors for various applications, including biosensors [28,29,32,67,68,69], and new generation of thermoelectric converters for energy applications [65,66]. Unlike most thermoelectric materials, whose ZT are small at low temperatures, the ZT of phosphorene is still substantial (ZT > 1), even at room temperature. For example, Lv et al. [70], basing on the results of semiclassical Boltzmann equation and DFT calculations, have shown that ZT value of phosphorene under strain can reached up to 1.65 at room temperature. This means that phosphorene is inexpensive and flexible thermoelectric material that can operate near room temperature.

More details on the properties of black phosphorus and phosphorene can be found in the reviews prepared by Lee et al. [23], Jain et al. [25], Khandelwal et al. [33], Yi et al. [27], Wu et al. [31], et al. [32], Li et al. [71], and Akhtar et al. [72].

### 2.2. Features of Black Phosphorus and Phosphorene Synthesis

#### 2.2.1. Bulk Black Phosphorus

Black phosphorus can be synthesized by a variety of methods [73]. But the simplest method for the synthesis of black phosphorus is to heat (without air access) white phosphorus at elevated temperatures under high pressure. In particular, Bridgeman [74] in 1914 conducted such a synthesis at a temperature of about 200 °С under 1.2 GPa within 5–30 min. Subsequently, in the 1980s, this process was improved, and BP single crystals with significantly improved quality and increased size were synthesized [75,76,77]. In particular, Maruyama et al. [75] developed a technology that allowed to synthesize black phosphorus in comparatively moderate pressure (0.5 MPa). To reduce pressure, Maruyama et al. [75] used the solution of white phosphorus and liquid bismuth heated to 300 °С. 

Currently, given the toxicity and flammability of white phosphorus, black phosphorus is mainly synthesized using red phosphorus as a raw material. However, for the transition of red phosphorus to crystalline black phosphorus it is required a higher pressure (2.5 GPa) and heating to *T* > 200 °C. At lower pressures, amorphous BP is formed. The transformation of red phosphorus into BP can be carried out at normal pressure (at *T* ~370 °C), but this requires the use of mercury as a catalyst, as well as a small amount of black phosphorus for the fuse [78]. Therefore, at the moment chemical vapor transport (CVT) method has become more widespread. According to this method, red phosphorus and additives of gold, tin, and tin (IV) iodide in small amounts are sustained under low pressure condition at a temperature of ~600 °C for several days. This method was proposed by Lange et al. [79] and improved by Nilges et al. [80] and Köpf et al. [81]. In particular, in contrast to the method developed by Lange et al. [79], Kopf et al. [81] succeeded to reduce the amount of side phases such as Au_3_SnP_7_, AuSn, or Au_2_P_3_. One should note that “low-pressure” method allowed to produce high quality BP [40].

It was also established that the phase transformation from RP to BP can take place under the high-energy mechanical milling (HEMM) condition, which under certain conditions can also provide high pressures and temperatures. Park et al. [16] reported the synthesis of BP by HEMM in closed space using steel balls and milling vessel for 54 h. Closed space and special atmosphere is necessary to protect against BP oxidation during processing. Since the mechanical milling method can only obtain BP powder with relatively lower crystallinity, this method is rarely used to synthesize bulk BP intended for preparation of 2D black phosphorous.

#### 2.2.2. Phosphorene

**Figure 4:**
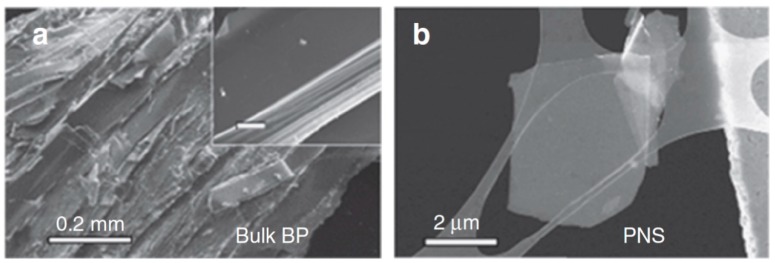
(**a**) Scanning electron microscopy (SEM) image of bulk BP. The inset is a magnified image showing the layered structure; scale bar, 10 μm; (**b**) SEM image of exfoliated BP nanosheets. Reproduced from Ref. [90]. Published by Macmillan Publishers Limited as open access, 2015.

The synthesis methods used to prepare 2D BP and phosphorene are listed in Table 2. One should note that the first 2D BP sheets consisting of few layers were prepared using the method of mechanical exfoliation, or scotch-tape-based microcleavage, widely used to obtain graphene and other 2D materials [17,54,73]. As mentioned above, BP is a kind of layered material and the interlayer interaction is controlled by weak van der Waals forces, which contributes to the successful application of mechanical exfoliation to obtain 2D BP. 2D BP nanosheets are then transferred on a Si/SiO_2_ substrate with following cleaning with acetone, isopropyl alcohol and methanol to remove any scotch tape residue. Typical SEM images of bulk BP and phosphorene are shown in Figure 4. In several studies such 2D BP sheets, fabricated by mechanical exfoliation, were used for development of electronic devices such as Field Effect Transistors (FETs) [19]. However, the experiment showed that mechanical exfoliation can produce only multi-layer 2D BP sheets that can contain up to 50 layers. Obtaining single-layer BP (phosphorene) using mechanical exfoliation failed. After numerous experiments, it was found that the thinnest forms of BP sheets obtained via mechanical exfoliation were bilayer flakes. Unfortunately, the reason of such effect is still unclear. It is suggested that multilayer BP flakes are easier accessed due to the strong inter-layer coupling. Lu et al. [82] have shown that this problem can be solved through the combination of mechanical cleavage and plasma ablation. They reported that when using Ar^+^ plasma, it is possible to thin few-layer BP (BP nanosheets) to monolayer phosphorene, i.e., to provide the necessary control of the number of layers in BP nanosheets. For these purposes they used Ar^+^ plasma (commercial 13.56 MHz RF source) with a power of 30 W and a pressure of 30 Pa. Repeating Ar^+^ plasma treatment was carried during 20 s at room temperature. It was established that the BP nanosheets after indicated treatment were very uniform. This shows that plasma thinning is a highly controllable technique for receiving homogeneous monolayer and few-layers phosphorene. In addition, 2D BP was also highly crystalline. Further, the effectiveness of the plasma treatment for the thickness control of BP flakes was also demonstrated by Jia et al. [83] and Lee et al. [84].

Although mechanical exfoliation is quite useful for fundamental research [91], the large-scale production of phosphorene using this method even in conjunction with Ar^+^ plasma treatment is still difficult. This approach to the formation of 2D materials is a labour intensive and not amenable to automation. In other words, this method suffers from low yield and a production rate that is not technologically scalable in its current form. In addition, this method does not provide reproducible parameters of 2D BP sheets, since this method does not have the ability to systematically control the shape, size and thickness, i.e., the number of the layers in the BP sheet. Another disadvantage is a residual organic contamination resulted from the adhesive tapes, which can have a significant effect on the surface properties of 2D BP. 

**Figure 5:**
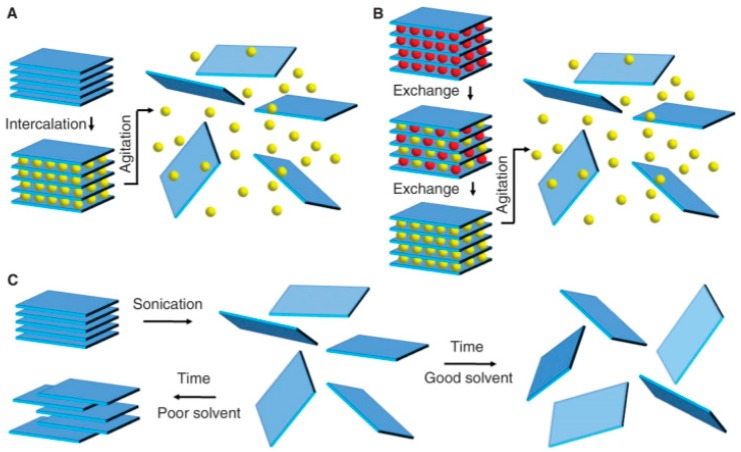
Schematic description of the main liquid exfoliation mechanisms: (**a**) ion intercalation; (**b**) ion exchange; (**c**) sonication-assisted exfoliation. Reproduced with permission from Ref. [97]. Copyright AAAS, 2013.

In order to avoid this limitation, a liquid phase exfoliation (LPE) was developed, and this method was reported as a reliable method to produce flakes of few- and single-layer BP in sizable quantities that can be processed by using existing industrial techniques, such as reel-to-reel manufacturing [92,93,94,95,96]. It should be borne in mind that liquid exfoliation is just a general concept, because there are different approaches to the implementation of this method. As is shown in Figure 5, the main methods of LPE include ion intercalation, ion exchange and sonication-assisted exfoliation [97]. However, among these methods, the most common in the formation of 2D BP is the method of sonication-assisted exfoliation. In this case the stable dispersions of 2D BP nanosheets are achieved via ultra-sonication, wherein collapsing cavitation bubbles yielded intense tensile and shear stress fields which break up the layered crystallites, and thus exfoliate, fragment bulk crystals of BP and produce exfoliated 2D BP nanosheets. As for ion intercalation, then in this method guest molecules, named inclusion complexes, are absorbed between the layers of materials [98]. These ionic species increase the space between the layers and weaken the boding between them, resulting in the reduction of the energy barrier needed for exfoliation. Therefore, after this intercalation, introducing even small energy between layers like thermal shock or ultrasonication can separate the sheets from each other, producing nanosheets. Ion exchange exfoliation works the same way (see Figure 5a,b). Examples of this approach to the formation of 2D BP are lithium [99] and sodium intercalations [100].

Basically, liquid exfoliation seems very simple. BP nanosheets can be made just by putting the bulk BP into some solvents and using proper ultrasound treatments. However, a lot of solvents are available for this process, so it is very important to choose a solvent, capable to provide 2D BP sheets of the required quality. How important it is to choose the right solvent is indicated by the fact that dispersants for graphene in organic solvents can degrade BP. For example, BP nanosheets are amorphized by ethyl cellulose, which is a conventional dispersant for highly concentrated graphene dispersions [101]. 

Currently, a large number of solvents have been tested for the preparation of BP nanosheets by liquid exfoliation [96]. In particular, Brent et al. [92] and Guo et al. [93] produced 2D BP nanosheets by 24 h sonication of bulk black phosphorus in a basic N-methyl-2-pyrrolidone (NMP) solution. The AFM results suggested that largest BP nanosheets, obtained in such solution, consisted of three to five layers. Yasaei et al. [94,95] tested several solvents such as alcohols, chloro-organic solvents, ketones, cyclic or aliphatic pyrrolidones, N-alkyl-substituted amides, and organosulfur compounds. These solvents with different properties covered a wide range of surface tensions and polar interaction parameters. Yasaei et al. [94,95] concluded that the aprotic and polar solvents like dimethylformamide (DMF) and dimethyl sulfoxide (DMSO) are most appropriated for the exfoliation and obtaining BP nanosheets. After 6-h sonication at 130 W for 0.2 mg bulk BP in 10 mL solvents (DMF and DMSO) and 30 min centrifugation at 2000 r/min, Yasaei et al. [94,95] obtained uniform and stable dispersions with concentrations of BP nanosheets up to 0.01 mg/mL. Kang et al. [101] also exfoliated 2D BP in various solvents such as acetone, chloroform, hexane, ethanol, isopropyl alcohol, dimethylformamide, and *N*-methylpyrrolidone and compared prepared suspensions. It has been established that the concentration of BP dispersions strongly depends on the solvent properties and monotonously increases with the boiling point and surface tension of the solvents. Kang et al. [101] also found that NMP was the best solvent for producing stable BP suspensions with the highest concentration of BP nanoflakes. Hanlon et al. [59] have shown that the solvent Ncyclohexyl-2-pyrrolidone (CHP) can also be used for this purpose. After sonication for 5 h and centrifuging the solution at 1000 r/min for 180 min, the concentration of BP nanosheets in CHP-based solution was increased up to 1 mg/mL. AFM studies showed that 70% of BP nanosheets obtained using this method had less than 10 layers. It is important to note that centrifugation of the dispersed BP solutions at different speeds is a method commonly used to separate exfoliated BP nanosheets by size.

As for the size of BP nanosheets, the dispersions, obtained by the LPE method, contained both very small flakes with an average length of ~130 nm, and very large flakes with an average length of ~2.3 μm [54]. The spread in the thickness of formed BP flakes is observed to the same extent. For example, in the dispersion used by Wood et al. [102] for the manufacture of devices based on BP, the thickness of BP flakes varied in the range of 10–150 nm. At that the content in the dispersion of flakes having only a few layers, is usually no more than a few tens of percent by quantity. Of course, this is a very large variation in the parameters of obtained BP nanosheets, and the content of ultrathin BP nanosheets in dispersion is too low for many applications. As a rule, this problem is solved by optimizing the composition of the solvents used for exfoliation. The results of these studies can be found in [103,104,105,106]. In particular, Xu et al. [104] reported that a phytic acid (C_6_H_18_O_24_P_6_) is a promising additive during LPE in DMF to prepare uniform and ultrathin BP nanosheets with a large size. The average size of BP flakes reached several tens of micrometers. Kang et al. [103] have shown that the surfactants 2% w/v sodium dodecyl sulfate, SDS, added in NMP can improve solution stability and produce thinner BP flakes. Kumar et al. [105] and Brent et al. [106] also reported that the addition of a surfactant, Triton X-100, made it possible to optimize the process and limit the oxidative degradation of BP.

The above discussions show that most solvents used for liquid exfoliation are common organic solvents. However, an experiment showed that the use of these solvents has significant drawbacks: organic solvents used for liquid exfoliation are toxic, they have a high boiling point, and it is difficult to remove these solvents. Therefore, it is quite natural that the development of methods that use water solutions for liquid exfoliation was of great interest. It should be noted that the resolving this problem is difficult to solve, since previous experimental studies have shown that mechanically exfoliated BP has extremely high sensitivity to moisture [32]. However, Wang et al. [107] reported that this task can be solved. Moreover, Wang et al. [107] found that the water-exfoliated BP nanosheets remained stable in water for weeks without obvious degradation in the dark and a noticeable change in the morphology of nanosheets.

Electrochemical exfoliation (ECE) is another top-down approach to the manufacture of 2D BP nanosheets. This method was successfully used by Erande et al. [87,108] for preparing an atomically thin BP nanosheets. It was shown that after applying a voltage between two electrodes, a bulk BP crystal fixed on a highly conductive metal electrode and a platinum wire, ^•^OH and ^•^O radicals are formed around the BP crystal. The radicals diffuse into the space between consecutive atomic layers of the BP crystal and weaken the van der Waals interaction between these layers. As a result, BP nanosheets are separated from the bulk BP crystal and are dispersed into the electrolyte solution. Later on, the same approach was used by Mayorga-Martinez et al. [109] to prepare BP nanosheets.

The experiment showed that the bottom-up methods such as chemical vapor deposition (CVD) and wet chemical synthesis can also be used for preparing 2D BP films [110,111,112]. One should note that CVD is typical bottom-up method, used to fabricate various 2D nanomaterials. The layers of the 2D materials were deposited from the gas phase containing the necessary precursor onto substrates being at high temperature. However, as compared with top-down methods, discussed above, the bottom-up methods used for preparing 2D BP layers are much less common. Although, chemical bottom-up methods have not made a breakthrough in deposition of high quality 2D BP layers, these methods, especially CVD, undoubtedly have a promising future, as with the development of phosphorene-based electronics and optoelectronics, the requirements for quality and controllability of the parameters of the 2D BP layers will increase. In addition, the CVD process provides good crystallinity and high purity of deposited phosphorene [112]. The above is an important advantage of the bottom-up methods such as CVD in comparison with chemical and electrochemical exfoliation. Despite the fact that large quantities of the 2D BP can be produced by LPE and ECE methods, this approach typically introduces defects or phase transformations in BP that degrade electronic properties. Thus, these methods are not suitable for fabricating high-quality phosphorene. In addition, the scalability of the LPE method is limited by the utilization of sonication as an energy source. 

Since the sonication can damage 2D flakes more or less, it was developed a gentle method which is a less energy-intensive shearing process for exfoliation in an organic solvent such as NMP [113]. Applied to 2D BP, this method has been adapted by Xu et al. [114]. More detailed description of mentioned above methods one can find in Refs. [25,26,27,31,33,72,73,91,96].

## 3. BP-Based Humidity Sensors

### 3.1. Achievements

**Figure 6:**
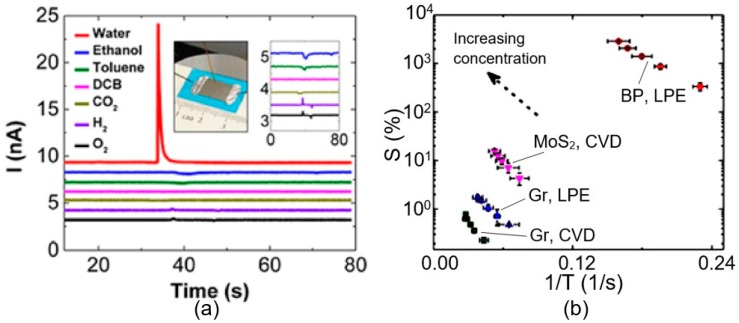
(**a**) The response of the BP sensor to different analytes. The inset (right) magnifies the same curves for clarity. The inset (left) shows the schematic of a typical BP-based sensor fabricated using principles of thick film technology; (**b**) comparison of sensitivities of a typical BP film, graphene and MoS_2_ sensors. (**a**) Reproduced with permission from Ref. [95]. Copyright American Chemical Society, 2015. (**b**) Reproduced with permission from Ref. [33]. Copyright Elsevier, 2017. Data from Ref. [94].

Numerous studies in recent years have shown that black phosphorus shows a great potential for humidity sensor applications. It was established that the best BP-based sensors have an ultrasensitive (Table 3) and selective response (see Figure 6) toward humid air with the trace level detection capability. Most of these sensors were fabricated using 2D BP sheets prepared by the method of liquid phase exfoliation. 

**Figure 7:**
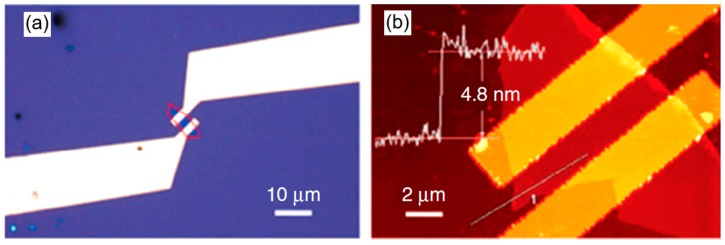
(**a**) Optical microscopy and (**b**) atomic force microscopy (AFM) images of the phosphorene nanosheet (PNS)-based FET sensor showing that the PNS electrically bridges the gold electrodes. The profile in (**b**) indicates the PNS has a thickness of 4.8 nm. Reproduced from Ref. [90]. Published by Macmillan Publishers Limited as open access, 2015.

**Figure 8:**
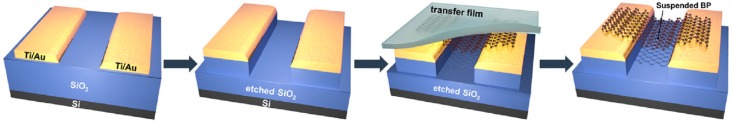
Schematic of fabrication of the suspended structure via dry transfer. Suspended BP flakes were fabricated on two high posts formed by the etched SiO_2_ layer. Reproduced with permission from Ref. [123]. Copyright Elsevier, 2017.

Typical view of the BP-based humidity sensors is shown in Figure 7. As a rule, these sensors had the configuration of field effect transistors (FET). For their fabrication various approaches can be used. For example, Figure 8 shows a schematic of the procedure for fabricating a device structure with suspended 2D BP layer. BP flakes mechanically or liquid phase exfoliated from the bulk BP crystals were transferred onto a SiO_2_/Si substrate with pre-patterned source-drain electrodes via a dry-transfer technique. A drop-casting method can also be used for sensor fabrication [115]. For these purposes the BP nanosheet dispersion is used.

#### 3.1.1. Resistive Humidity Sensors

Sensors that most clearly demonstrate the advantages of the BP-based humidity sensors were developed by Yasaei et al. [94,95]. BP films used in this study were prepared using a liquid phase exfoliation in DMF or DMSO solutions. Electrical contacts to 2D BP film were fabricated using gold paste or gallium-indium eutectic. The humidity sensing characteristics of the fabricated devices, shown in Figure 6a, were tested in a dynamic mode using the pulse injection method. It is seen that nearly, a 5-fold increase in the drain current was observed upon injection of water vapor in measurement cell. It is important that the response to other vapors and gases was much smaller. As it is seen in Figure 6a, indicated gases induced a negligible conductivity response in the sensor. This selective response of BP-based sensors towards water vapor distinguishes them from most solid-state humidity sensors and makes them very promising for humidity detection in real conditions, as they overcome cross-sensitivity and false response caused by the appearance of toxic and flammable gases and vapors in the atmosphere. Although it must be recognized that, despite its higher sensitivity to humidity, BP-based sensors can detect toxic gases in the atmosphere, such as NO_2_ and NH_3,_ at few ppm [69,124]. BP-based humidity sensors also showed fast recovery within 1–5 s after water vapor injection, which is also important for real-time detection of gas or air humidity. 

**Figure 9:**
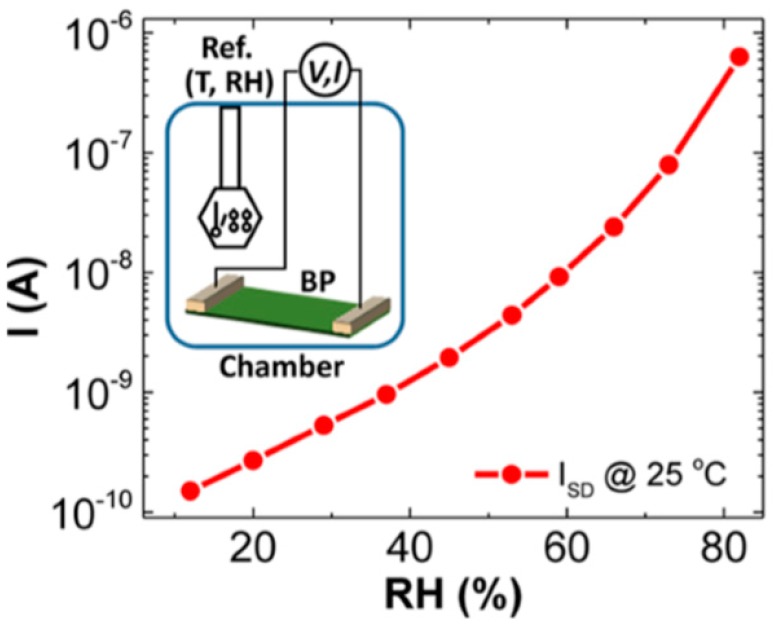
The current of a typical BP sensor measured at fixed voltage versus RH at 25 °C. The inset shows the scheme of the custom-made chamber that is used for the experiment. Reproduced with permission from Ref. [95]. Copyright ACS, 2015.

Yasaei et al. [94,95] also compared the conductivity response of 2D BP, graphene and 2D molybdenum disulphide (MoS_2_) based humidity sensors. Figure 6b shows the responses S (where S = (I − I_0_)/I_0_, %) of these sensors with respect to reciprocal recovery time (1/t_rec_) upon injection of water vapor, measured in steady state conditions. It is seen that under identical conditions, BP sensors exhibited higher sensitivity (by 2 orders of magnitude) and faster recovery process (2-fold faster) compared to graphene and MoS_2_-based humidity sensors. It is also important to note that, in terms of the sensitivity the 2D BP sensors are not inferior to the best samples of solid-state humidity sensors [12]; the conductivity of BP film increased by ~4 orders of magnitude as the relative humidity varied from 10%RH to 85%RH (see Figure 9). 

Erande et al. [80] also fabricated conductometric humidity sensor using stacked 2D BP flakes prepared by electrochemical exfoliation method. However, the response of these sensors was small in comparison with data reported by Yasaei et al. [94,95]. The resistance of the BP-based sensor decreased by ∼85% with increasing humidity from 11%RH to 97%RH. The response time and the recovery time were also longer, ∼101 s and ∼26 s, respectively. It should be noted that mentioned above humidity sensors had quite thick BP films (a few μm), which probably is not optimal for achieving maximum sensitivity to humidity. This conclusion can be made from the results obtained by Late [116]. Late [116] studied the humidity sensing characteristics of BP nanosheet based films with different thickness. He prepared three kinds of BP films by liquid phase exfoliation and found that the BP sample with smaller thickness exhibited both better response to humidity, and faster response and recovery processes. 

**Figure 10:**
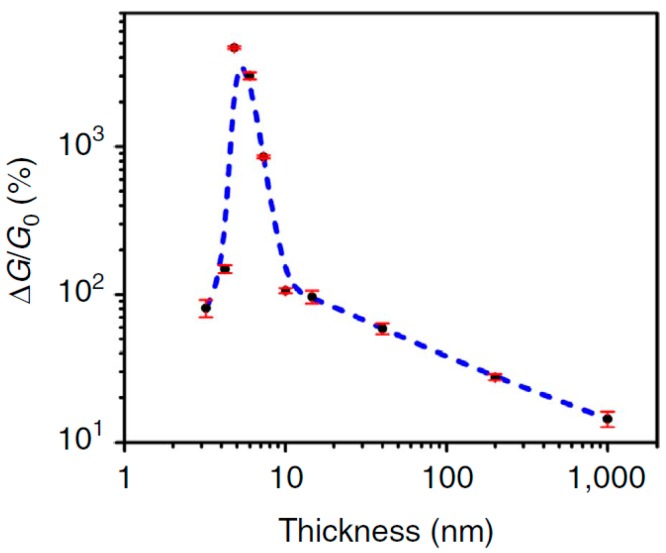
The influence of phosphorene nanosheets thickness on the conductivity response to NO_2_ (500 ppb) in dry air. Reproduced from Ref. [90]. Published by Macmillan Publishers Limited as open access, 2015.

Low sensitivity of sensors developed by Erande et al. [87], may also be associated with the features of the structural parameters of 2D BP flakes, prepared by electrochemical exfoliation and used in the manufacture of sensors. Such a parameter, in particular, can be not the total thickness of the BP film, but the thickness of the 2D BP flakes forming these films. Such an assumption is quite real. For example, Cui et al. [90], testing the BP-based NO_2_ sensor found that the sensitivity of this device dramatically increased when the thickness of BP nanosheets became smaller than ~10 nm. At that the greatest increase in the sensitivity was observed with decreasing thickness of BP nanosheets to 4.8 nm, after which the sensitivity dramatically decreased (Figure 10). Such behavior Cui et al. [90] explained by the effect of nanosheet thickness, i.e., the number of the layers in the BP nanosheets, on the band gap of 2D BP (see Figure 2 and Figure 3). Physically, this dependence arises from the fact that the ability to adsorb gas molecules and affect the conductivity depends on the band gap and concentration of the charge carriers in material. Therefore, there should be an optimum band gap range for achieving maximal sensor effect. 

It was established that besides BP flakes or nanosheets, BP quantum dots (QDs) can also be used to fabricate humidity sensor. Zhu et al. [117] have shown that BP QDs can be prepared fast enough by liquid phase exfoliation method using a house-hold kitchen blender. BP QDs-based humidity sensors were fabricated on the substrate with interdigital electrodes using spin-coating technology. It is important to note that these sensors also had extremely high sensitivity to air humidity. The resistance of the sensor decreased by ∼4 orders of magnitude with RH varied from 10% to 90%. More importantly, the response of the sensor was stable. Only a slight change in the sensor response was observed after 66 h operation in the range of high humidity level (90%RH), and negligible change was found in the range of moderate humidity level (35%RH).

As for the mechanisms explaining the sensitivity of BP to air humidity, then, as a rule, they do not differ from the mechanisms developed for metal oxides and other solid state materials [12,13,87]. According to this mechanism, multilayer physical adsorption of water molecules generates protons, leading to a decrease in electrical impedance of BP-based structures.

#### 3.1.2. Other Types of Humidity Sensors

**Figure 11:**
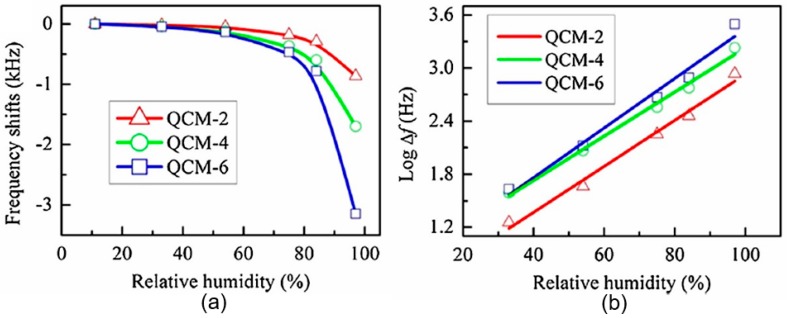
(**a**) The frequency response of the black phosphorous based QCM humidity sensors as a function of humidity; (**b**) the logarithmic fitting curve of Log (Δf) versus humidity for all the sensors. QCM-2, QCM-4 and QCM-6 indicates sensors with 2, 4, and 6 μL of BP nanosheets deposited on the electrode of QCMs. Reproduced with permission from Ref. [115]. Copyright Elsevier, 2017.

Yao et al. [115] have shown that the quartz crystal microbalance (QCM) humidity sensors based on 2D BP can also be developed. A QCM with deposited sensing film is an excellent and proven mass sensitive platform that can transform the adsorbed molecular mass to a frequency-dependent signal. The 2D BP nanosheets, used as sensing layer in QCM humidity sensors, were synthesized by traditional liquid exfoliation method. BP nanosheets dispersion was deposited on the surface of QCM with a 10 MHz fundamental frequency using a drop-casting method. From Figure 11 it is seen that the resonance frequency decreases as the humidity is increased in the range of 11.3%RH–97.3%RH. At that the resonance frequency response of the BP-based QCM sensors with respect to humidity exhibited a well-defined logarithmic curve. Moreover, the sensitivity of the sensor was strongly proportional to the amount of BP nanosheets deposited on QCM. The response time of mentioned above BP sensors also depended on the amount of sensing material deposited on the surface of QCM, i.e., on the thickness of humidity sensitive layer. As the BP thickness of the film increased, the response time increased from 14 s to 29 s. At the same time, the recovery time of sensors was almost independent of the thickness of the sensitive layer and was equal to ~10 s. It is important that the moisture hysteresis for this sensor was observed to be under 4%. Yao et al. [115] have also shown that the BP-based QCM sensors exhibited high stability over the wide range of relative humidity. It was reported in Ref. [96] that developed sensors were stable during 4 weeks even at a high humidity level 97.3%, and the drift, similar to a conductivity drift observed by Yasaei et al. [95], has not been observed. How it was established by Yasaei et al. [95], the BP conductometric sensor exhibited a good stability without obvious drifts in its sensing characteristics after 3 months’ exposure to atmosphere only with relatively low humidity (25 °C and 25 ± 12%RH). At higher humidity, the drift of the characteristics of conductometric sensors becomes too pronounced. These results show that, compared with conductivity, the change in weight due to the physical adsorption of water vapor is not so sensitive to the degradation of phosphorene that occurs when interacting with oxygen and water vapor. This means that the QCM-based BP sensors are more suitable for humidity detection in the wide range of RH in comparison with conductometric-based BP sensors.

**Figure 12:**
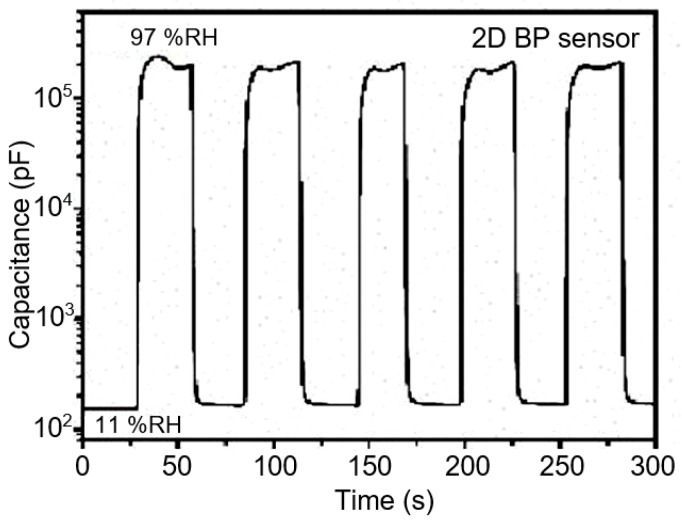
The capacitance response and recovery behavior of the BP humidity sensor when humidity changes between 11%RH and 97%RH. All measurements were conducted at 100 Hz and with a bias voltage of 0.5 V. Reproduced with permission from Ref. [122]. Copyright RSC, 2018.

**Figure 13:**
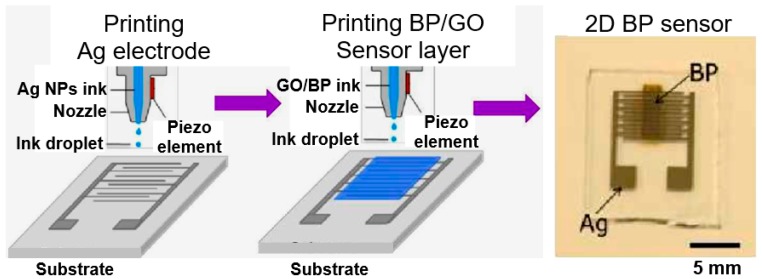
Schematic representation of the fabrication process of sensor device using inkjet printing technology. Reproduced with permission from Ref. [122]. Copyright RSC, 2018.

BP-based capacitance humidity sensors, developed by He et al. [122], also demonstrated high sensitivity. As the relative humidity level increased from 11% to 97%, the response (∆C/C) of the BP sensors increased by four orders of magnitude at 10 Hz frequency (see Figure 12). Moreover, the measured response and recovery time of BP sensors were within a few seconds. In accordance with general views on the operation of capacitive humidity sensors, the increased capacitance of BP films in humid air can be attributed to extensive adsorption and condensation of water molecules, which strengthens the polarization and increases the dielectric constant of the BP film [6,7,11,12]. The BP nanoflakes used for fabrication of capacitance sensors were prepared by LPE method. The average thickness of BP flakes was around 8.5 nm, which corresponds of approximately 10 atomic layers. Sensors were fabricated using inject printing technology. The design and technology used to manufacture the sensors are shown schematically in Figure 13. 

Miao et al. [125] also manufactured and tested capacitive BP-based humidity sensors. However, these sensors did not show such high sensitivity. Apparently, this is due to the features of the humidity-sensitive layers used in these sensors. Unlike He et al. [122], who used the principles of thick-film technology in the formation of the sensitive layer (*d* ~few micrometers), Miao et al. [115] in their sensors used few-layer BP flakes that were mechanically exfoliated from bulk BP crystals using a scotch tape and transferred to heavily doped and oxidized Si wafers. In such thin structures (*d* < 5 nm), it is difficult to realize capillarity condensation of water vapor, which often makes the maximum contribution to the change in capacitance of the tested structures.

Another type of BP-based humidity sensors was developed by Chen et al. [119]. Chen et al. [119] fabricated a BP-based passive microwave substrate-integrated waveguide (SIW) resonator humidity sensor operated at microwave frequencies (*f*_0_ ~3.6 GHz). For this purpose, they used commercial BP dispersion (1 mg/mL, dissolved in ethanol). The size of BP flakes was about several tens of micrometer. BP solution was deposited on the sensing slots using a drop-coating method. It was shown that the sensitivity of the SIM resonator humidity sensor with BP sensing layer, tested in the humidity range of 11–97%RH, was 197.67 kHz/%RH, about 40 times larger than that of the sensor without BP sensitive layers. Chen et al. [119] believed that this approach is promising for fabrication of low-cost passive RFID (Radio Frequency Identification) humidity sensor tag for environmental monitoring.

### 3.2. Limitations of BP-Based Humidity Sensors

#### 3.2.1. The Tunability of Black Phosphorus Properties

**Figure 14:**
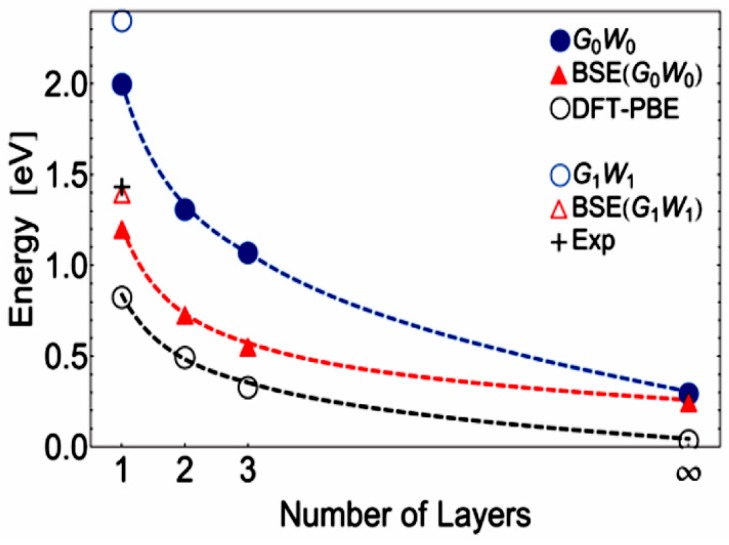
Band gap with increasing number of layers are calculated by various methods and fitting curves are presented with dashed lines: DFT—Density Functional Theory; PBE—Perdew–Burke–Ernzerhof functional; BSE—Bethe–Salpeter equation. The experimental value is brought from Ref. [67]. Reproduced with permission from Ref. [129]. Copyright American Physical Society, 2014.

No doubts that black phosphorus has unique humidity-sensitive properties, which makes this material promising for use in humidity sensors. But at the same time, black phosphorus has a number of disadvantages, which significantly limit its use in real devices. These include the technological difficulties of forming humidity-sensitive layers with a required thickness of BP nanosheets, and a strong dependence of the BP properties on the synthesis and subsequent processing conditions as well as on the layer thickness [23,51,125,126,127,128]. The first evidence of this dependence was presented in Figure 2 and Figure 3. Figure 14, representing the results of other theoretical calculations, also demonstrates the strong effect of the thickness of BP nanosheets on the value of the band gap. It can be seen that, regardless of the calculation method used, the band gap decreases as the number of layers in BP nanosheets increases from monolayer (phosphorene) to bulk phosphorous. In particular, from ab initio calculations with the GW approximation, the band gap of black phosphorus varies from 2 eV for monolayer to approximately 0.3 eV for bulk [129]. It is believed that the dependence of the band gap on the thickness is due to the charge carriers’ quantum confinement effect in the out-of-plane direction. Transition metal dichalcogenides (TMDCs) also have band gaps that vary with thickness, but this change is not so strong as in black phosphorus [130]. With a change in the thickness of BP nanosheets, their adsorption properties also change. In particular, the thickness-dependent band gaps and conduction band edges lead to different surface oxygen concentrations on the thinner and thicker BP [131]. Island et al. [15] have also found that the thinner flakes absorb water faster than the thicker ones. 

Rodin et al. [132] and Wang et al. [133] suggested that the strain can also induce a modification of the band gap. By the Density Functional Theory and the Tight-Binding Theory for monolayer black phosphorus, they have shown that as the strain increases, the original direct band gap of black phosphorus changes into an indirect band gap and, at last, it shows the zero band gap, peculiar to metal. The same result was obtained by Liu et al. [54] They reported that a compressive strain of 5% triggered in BP the transition from direct to indirect band gap. Liu et al. [54] also established that in case of negative strain, both armchair and zigzag direction strains lead to the band gap decrease. On the other hand, when positive strain is applied, the strain in the zigzag direction successively increases the band gap, while the band gap abruptly decreases with more than 5% armchair direction strain. According to Dai and Zeng [67], stacking pattern of black phosphorus is another factor that influences the band gap. Dai and Zeng [67] have found that depending on stacking pattern of BP, calculated band gaps of bilayer black phosphorus varied in the range of 0.8 eV–1.05 eV. 

**Figure 15:**
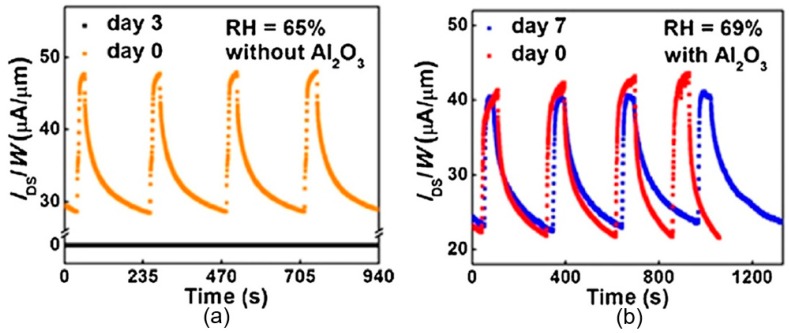
Long-term stability of the BP humidity sensors: (**a**) dynamic sensing response for the unencapsulated BP sensor right after fabrication (orange) and after being stored in ambient for 3 days (black); (black line RH ~21%, orange line RH ~65%); (**b**) dynamic sensing response for the encapsulated BP sensor right after fabrication (red) and after being stored in ambient for 7 days (blue) (blue line RH ~21%, red line RH ~69%). Reproduced with permission from Ref. [125]. Copyright American Chemical Society, 2017.

**Figure 16:**
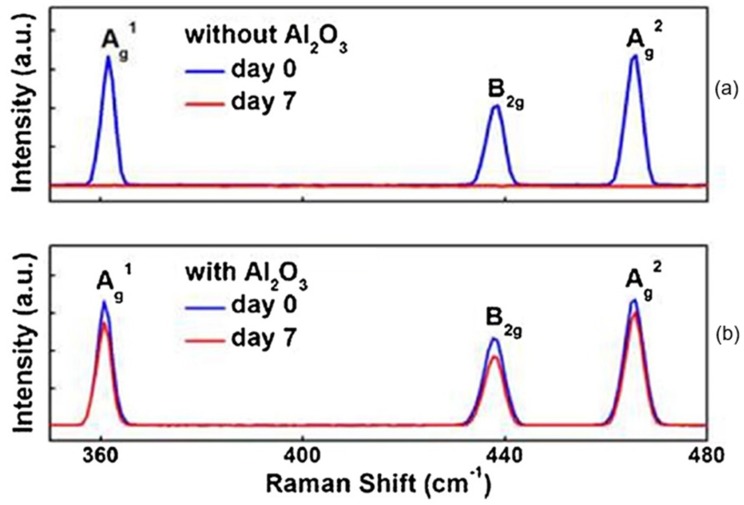
(**a**) Raman spectra of a BP flake without Al_2_O_3_ capsulation after exfoliation (blue line) and after 7 days under ambient condition (red line). (**b**) Raman spectra of a 6-nm-thick Al_2_O_3_ encapsulated BP flake immediately after exfoliation (blue line) and after 7 days under ambient condition (red line). Reproduced with permission from Ref. [125]. Copyright American Chemical Society, 2017.

**Figure 17:**
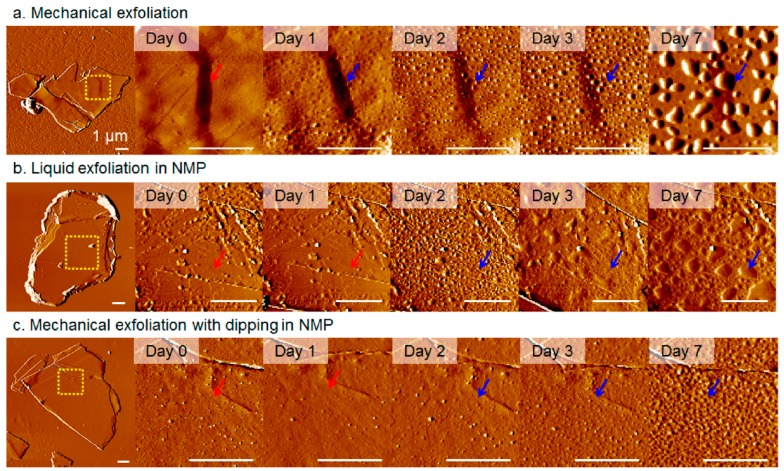
AFM amplitude images (amplitude scale: 5 × 5 nm (top left), 1 × 1 nm (magnified images)) of BP flakes prepared by (**a**) mechanical exfoliation, (**b**) solvent exfoliation in N-methyl-2-pyrrolidone (NMP), and (**c**) mechanical exfoliation followed by 1 h submersion in NMP. The leftmost image shows the entire flake, and the images progressing to the right show magnified views immediately after exfoliation up to 7 days in ambient conditions. Structural deformations (i.e., apparent bubbles) are observable on the mechanically exfoliated sample after 1 day and on the rest of the samples after 2 days. Red and blue arrows indicate the same position on the BP flake before and after the appearance of bubbles, respectively. All flakes are thicker than 150 nm, and all scale bars are 1 μm. Reproduced with permission from Ref. [101]. Copyright ASC, 2015.

Thus, taking into account the strong interrelation of the electronic parameters of the phosphorene with its adsorption and catalytic properties, it becomes clear that all of the above makes it difficult to manufacture phosphorene-based sensors with reproducible parameters. But the main disadvantage of black phosphorus, limiting its use in humidity sensors, is still the instability of their parameters [29,31,33,59,134]. It was found that if bulk BP was stable at atmospheric conditions for a few months, then exfoliated 2D BP showed a relatively high reactivity and strong air instability [135]. After a few days, the BP flakes degraded, and large water droplets were observed on their surface. As a result, the sensors ceased to function (see Figure 15a). In addition to the sharp deterioration in the sensor response, the degradation of BP after continuous exposure in the air was accompanied by a gradual decrease of the Raman peak intensity [125,136]. Typical Raman spectra are shown in Figure 16a. The observed change in Raman spectra is usually associated with amorphization of BP and irreversible conversion of BP into PO_x_ compounds [102]. The degradation of BP also manifested itself on the images obtained with atomic force microscopy (AFM). AFM images of BP nanosheets prepared by various methods at various stages of degradation are shown in Figure 17. Kang et al. [101] have shown that despite better stability of the properties of BP nanosheets prepared by solvent exfoliation in NMP, after 7 days, larger and higher bubbles were observed on all BP nanosheets regardless of how they were prepared.

#### 3.2.2. Instability of Black Phosphorus

**Figure 18:**
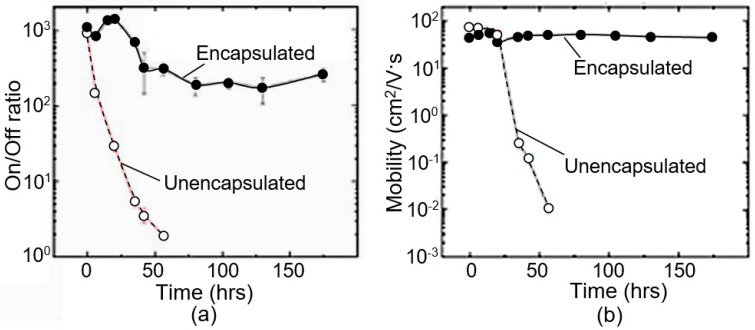
On/off ratio and the hole mobility of the black phosphorus field effect transistor for encapsulated one and unencapsulated one (**a**,**b**). Reproduced with permission from Ref. [102]. Copyright American Chemical Society, 2014.

Currently the degradation mechanism of 2D BP is controversial and not entirely clear [25,31]. In most previous studies, researchers simply attributed the degradation of 2D BP in ambient conditions to moisture and strong hydrophilicity. However, the experiment showed that there are more factors leading to instability of the parameters of black phosphorus. But the most important are degradations caused by oxygen, light, water and temperature [23,137,138]. In Figure 18, it is possible to identify the progress of the degradation of black phosphorus in the ambient condition. Given the specifics of the work of humidity sensors, the presence of degradations caused by oxygen and water molecules are especially critical for their performance.

The interaction with oxygen has been established by both calculation and experiment. It was found that the dissociation of O_2_ on black phosphorene is endothermic with an energy of −4.07 eV. At that the calculated dissociation barrier of O_2_ on phosphorene is only 0.54 eV [138]. This means that during interaction, the O_2_ molecule easily dissociates and chemisorbs at the surface of black phosphorus already at room temperatures [138,139]. It was also established that as a result of this reaction, in addition to chemisorbed oxygen, interstitial oxygen may appear in the BP lattice, which along with chemisorbed oxygen influence the properties of black phosphorus devices, initiating their degradation. In particular, interaction with oxygen initiates huge structural deformation and the appearance of deep donor/acceptor levels in the band gap of black phosphorus, accelerating the recombination of charge carriers at the surface of black phosphorus [139]. 

According to Wang et al. [138], H_2_O will not strongly interact with pristine phosphorene, however, an exothermic reaction with H_2_O could occur if phosphorene is first oxidized. It was established that black phosphorus with chemisorbed oxygen has strong affinity for water molecule. As a result, BP acquires increased hydrophilicity, which creates the necessary conditions for degradation by water molecule. For example, it was found that the strong dipole–dipole interaction between water molecule and phosphorus causes a significant distortion in the black phosphorus structure. In particular, computational studies by Hanlon et al. [59] showed that the degradation, occurred by the reaction of water molecules with BP, causing the removal of phosphorus atoms and leading to the formation of phosphine and phosphorous acid. 

**Figure 19:**
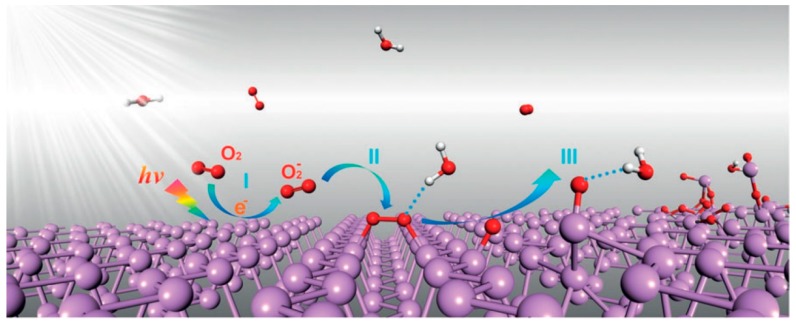
The light-induced ambient degradation process of phosphorene (Ph). Step 1: O_2_^−^ is generated through a charge transfer reaction under ambient light (O_2_ + hν → O_2_^−^ + h^+^; where h^+^ is a hole); step 2: O_2_^−^ dissociates at the surface and forms two P–O bonds with the phosphorene (O_2_^−^ + Ph + h^+^ → P_x_O_y_); step 3: through the hydrogen-bond interaction, water molecules draw the bonded O and remove P from the surface and break the top layer of phosphorene. Reproduced with permission from Ref. [131]. Copyright Wiley-VCH Verlag, 2016.

At the same time, Zhou et al. [131] believe that the oxidation alone does not break down the structure of phosphorene. Wang et al. [138] also believe that the oxidation of BP followed by exothermic reaction with water is the most likely route for the chemical degradation of phosphorene-based devices in the air. Based on ab initio calculations, Zhou et al. [131] came to the same conclusion. They suggested a three-step 2D BP ambient degradation mechanism. First, superoxide anions (O_2_^−^) are generated via a charge transfer reaction on the 2D BP surface under ambient light. This means that the light illumination is the trigger of the degradation process of phosphorene in ambient conditions. Then, the dissociated O_2_^-^ species react with phosphorus on the BP surface to produce two P–O bonds. Finally, water molecules interact with the bonded oxygen through hydrogen-bond interactions to remove the phosphorus clusters and break the top phosphorene layer. This mechanism is schematically shown in Figure 19. In addition, Island et al. [15] have shown that at black phosphorus aging in the air, containing water vapor, its volume during several days increased up to 200%.

Another degradation mechanism was identified by Island et al. [15]. They have established that long term exposure of BP to ambient conditions resulted in a layer-by-layer etching process of BP flakes, which at a certain stage led to the formation of a single layer of BP, phosphorene. Such a change in the thickness of the layer is undoubtedly a noticeable factor influencing the parameters of the devices. Such a change, in particular, was observed when testing BP FETs [15]. Continuous measurements of BP FETs in the air have shown the degradation and break-down of the channel material after several days. Etching process is explained by the formation of phosphoric acid during oxidation, which is further removed by evaporation either in ambient environment or when the sample is placed in vacuum for measurements [140].

Studies established that light and temperature also contribute to the degradation of 2D BP. Favron et al. [136] have found that with the oxygen and water existent condition, 2D BP are also subject to photoinduced degradation. Moreover, the degradation rate was proportional to the oxygen concentration and light intensity. 

As for thermal stability of the two-dimensional black phosphorus, the studies made by Liu et al. [141] have shown that the decomposition of 2D BP was observed at ~400 °C in vacuum, in contrast to the 550 °C bulk BP sublimation temperature. As a result of decomposition, black phosphorous is being transformed into amorphous red phosphorus.

## 4. Is It Possible to Improve the Stability and Reproducibility of BP Parameters?

**Figure 20:**
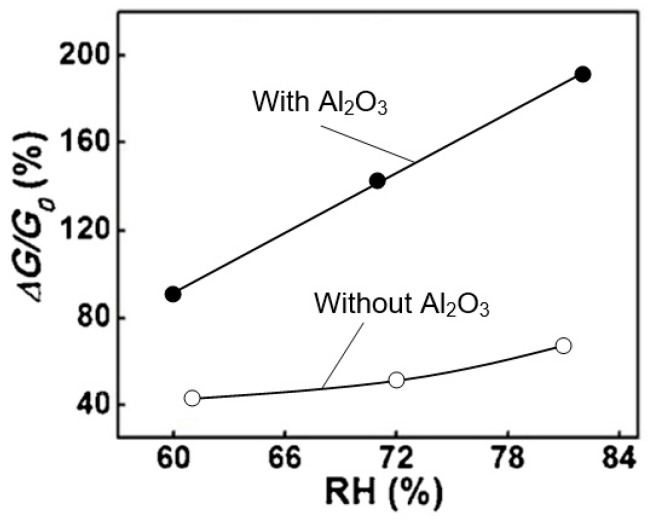
Conductivity response of the BP humidity sensors without and with Al_2_O_3_ layer as a function of RH levels. Reproduced with permission from Ref. [125]. Copyright American Chemical Society, 2017.

In recent years, many studies have been conducted aimed at stabilizing the properties of black phosphorus [31,142]. In particular, to prevent detrimental effect of the degradation, Wood et al. [102] and Miao et al. [125] made the passivation on the black phosphorus layer by the AlO_x_ overlayer. Encapsulation layers such as h-BN [126], polymer [143], SiO_2_ [144], MoS_2_ [145], graphene oxide (GO) [146], and graphene [126] were also applied as barriers to protect 2D BP from its structure and chemical degradation. As we can see in Figure 13 and Figure 14, passivated (encapsulated) black phosphorus maintains its properties well compared with unencapsulated one. For example, according to Miao et al. [125], the BP device encapsulated a 6-nm-thick Al_2_O_3_ layer did not display any noticeable change after being stored in ambient condition for over 5 days. Wood et al. [102] claimed that AlO_x_ overlayers deposited on the BP effectively suppress the degradation of encapsulated BP FETs during two weeks. Thus, it seems obvious that passivation on the black phosphorus layer blocks the fast degradation by oxygen and water molecule. However, if this approach gives good results for field-effect transistors, then the surface passivation has limited application for humidity sensors. For gas sensing applications, BP has to be in direct contact with the environment. Otherwise, with full sealing, the parameters of the sensors will be determined by the properties of the capsulation coating, and not black phosphorus. Such situation may be accompanied by a sharp drop in sensitivity. In the case of BP-AlO_x_ layer such a strong drop in sensor response is not observed (see Figure 20). But this is understandable, since it is difficult to imagine complete BP encapsulation by so thin AlO_x_ layer (*d* ~6 nm). 

A different approach to improving the stability of BP in the air has been developed by Li et al. [142]. They suggested to use ionophore for BP encapsulation. Ionophore solution was spun coated on the surface of BP flakes, mechanically exfoliated by scotch tape based method and transferred onto the SiO_2_/Si substrate. It was established that ionophore film effectively reduced negative influence of ambient environment on the parameters of BP layers. As a result, high performance BP sensors with significantly improved stability were developed. The ionophore-encapsulated BP devices were still in good shape after 1 week of ambient exposure, with source-drain current I_DS_ variation less than 10%. However, currently, this approach has been implemented only in the manufacture of BP devices aimed for ion sensing. Li et al. [142] reported that the BP sensors were able to detect heavy metal ions over a wide range of concentration. How to implement this approach in relation to humidity sensors is not yet clear, as used by Li et al. [142] ionophore films effectively reduced the contact between BP flakes and water molecules, which should be detected.

For sensor fabrication Abbas et al. [20] suggested to use thicker BP flakes (*d* ~55 nm) that are not subject to such rapid degradation. But even in this case, the time of stable operation is very limited and does not meet the requirements for the devices intended for the sensor market. As noted earlier, even bulk BP is stable at atmospheric conditions for only a few months. In addition, such sensors, as mentioned earlier, should have reduced sensitivity, which is another factor limiting the use of thick BP flakes to develop humidity sensors.

**Figure 21:**
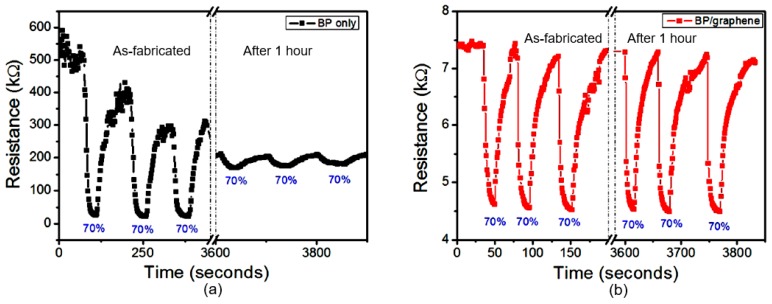
Transient response and estimated stability of the humidity sensor after 1 h based on (**a**) BP only and (**b**) BP/graphene heterojunction. Reproduced from Ref. [118]. Published by Springer Nature Publishing AG as open access, 2017.

**Figure 22:**
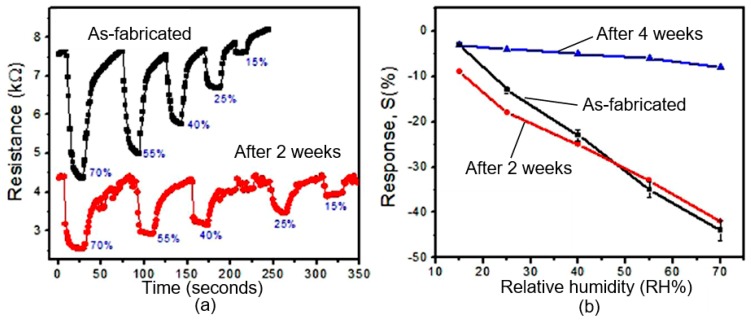
(**a**) Transient response of the BP/graphene humidity sensor as-fabricated and after 2 weeks. (**b**) Aging influence on sensor response of BP/graphene humidity sensor. Reproduced from Ref. [118]. Published by Springer Nature Publishing AG as open access, 2017.

A recent approach to overcome the difficulty related to the degradation of BP under ambient conditions has been reported by Tan et al. [147]. They suggested to intercalate alkali metal hydride in multi-layered BP to improve the stability of BP. The XPS, LEED, chemical, and transport studies of synthesized quasi-monolayer LiH-BP revealed that LiH intercalation had really reduced the reactivity of BP towards oxygen. The stabilizing effect was achieved by donating electrons to electron trap sites in BP and neutralizing the hole oxidizers in BP. Moreover, the LiH-BP hold good crystallinity and showed carrier mobility up to ~800 cm^2^/V·s even after ambient exposure. AFM and Raman studies have also shown the stability of intercalated BP after exposure to the surrounding atmosphere for one month. However, these studies do not provide an answer to the question—how will such structures behave in humidity sensors? With this regard, the studies conducted by Phan et al. [118] give a more specific answer to this question. They have shown that the stability of the BP-based humidity sensor can be significantly improved through the formation of BP/graphene hybrid structures (see Figure 21). As it is seen, the sensor based on BP-graphene heterostructure had good repeatability and non-degradation after 1 h. However, these sensors are also a subject to aging with longer operation. As can be seen in Figure 22b, the sensor had a significant decrease in response after 4 weeks. In this study, a large quantity of BP materials was synthesized in a powder form with a technique of high energy ball milling (HEBM). BP/graphene heterojunction was formed on the graphene surface using an electrospray deposition of BP layer. The wafer-scale of single layer graphene was synthesized by chemical vapor deposition. It should be noted that the reasons for the increased stability of such sensors are not clear. Phan et al. [118] believe that chemical bonding between phosphorus and carbon (P–C) play a significant role in stabilizing BP/graphene composites/ hybrids. However, it is more likely that the preservation of the functional properties of such sensors is not due to the greater stability of the BP layer and interface in BP/graphene hybrid structures, but due to the presence of graphene in these structures, which retains sensitivity to water vapor even with the degradation of the properties of black phosphorus. But it should also be noted that the presence of an additional layer of graphene at the same time is the reason for the decrease in the value of the sensor response for as-fabricated devices. The highly conductive graphene layer shunts a higher and more sensitive 2D BP layer.

**Figure 23:**
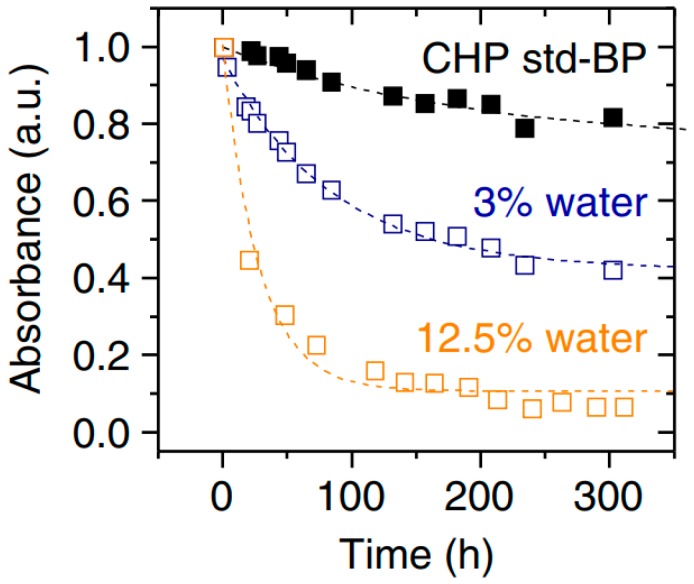
The stability of absorbance (465 nm) by black phosphorous nanosheets exfoliated in different conditions: 1-Fitted time-dependent data for BP nanosheets exfoliated in CHP and 2, 3 data for BP exfoliated in CHP solution with 3 vol% and 12.5 vol% of water added. Reproduced from Ref. [59]. Published by Macmillan Publishers Limited as open access, 2015.

Studies have also shown that to preserve the properties of BP flakes, they must be stored in solutions. For example, Kang et al. [101] established that 1 h submersion of BP nanosheets exfoliated mechanically in N-methylpyrrolidone (NMP) increased the stability of their properties. However, even in this case, the BP flakes change properties, albeit at a slower rate. According to Hanlon et al. [59], the most stable characteristics were performed by the BP nanosheets exfoliated in dry, deoxygenated N-cyclohexyl-2-pyrrolidone (CHP). The BP exfoliated in the aqueous environment (CHP + H_2_O) degrades most rapidly (see Figure 23). However, even in this case, the degradation of the characteristics was much slower than in the case of BP nanosheets exfoliated micromechanically.

As for the approaches that are really aimed at improving the reproducibility of the parameters of humidity sensors through the development of technology for obtaining BP flakes with identical controlled parameters, unfortunately such works do not exist. The experiment showed that most of the technologies used for the formation of 2D BP, give a fairly large variation in thickness, and, consequently, in the properties of BP. It was found that through centrifugation of a solution containing BP flakes, it is possible to reduce the spread in the parameters of BP flakes [26,148]. But to achieve a significant reduction in the dispersion in the thickness of BP flakes is very difficult, as the separation during centrifugation is affected by the weight of BP flakes, which depends not only on the thickness, but also on the size of these BP flakes. Therefore, at the moment, the most promising direction for the formation of BP flakes with a given thickness is the approach based on the use of the technology of chemical vapor deposition (CVD) [149]. For example, Smith et al. [88] reported on the CVD method for growing two-dimensional BP flakes of a large area (S > 3 µm^2^) with thickness corresponding to four layers. However, despite the fact that this work showed some perspective in the production of CVD-processed BP flakes with fixed thickness, further reports on the application of CVD BP flakes in humidity sensors are missing.

Certainly, a significant decrease in the dependence of sensor parameters on the BP flake thickness can be achieved by increasing the flake thickness itself. However, as was shown in Section 3.1, this will be accompanied by a decrease in the sensitivity of the sensors, which significantly reduces the attractiveness of 2D BP for the development of humidity sensors.

There is also an assumption that the increased stability of the 2D BP-based humidity sensors can be achieved by using phosphorene oxide [133,140,150], which, by analogy with graphene oxide should be more stable at ambient conditions. Really, simulations carried out for phosphorene oxide by Zhou et al. [131] have shown that the H-bonds between H and O keep the adsorbed H_2_O near the surface, but it does not break any bond, and the oxide monolayer maintains its integrity during the simulation time. The high stability of the fully oxidized phosphorene is derived from the formation of bridge P–O–P bonds. This is consistent with the experimental observation of stable oxide at the surface of bulk BP [140]. It was found that the majority of the oxides is made up of phosphorus pentoxide, which represents the most thermodynamically stable oxidation pathway. According to Edmonds et al. [140], phosphorus oxide only forms at the top layer of bulk black phosphorus and offers great potential as a stable and protective capping layer

However, Zhou et al. [131] have found that improved stability is observed only for fully oxidized phosphorene. For partial oxidized phosphorene without the formation of P–O–P bonds, the adsorbed H_2_O distorts the O-covered surface and leads to the break of P–P bonds in a short time. This means that the structural collapse of few-layer BP in the ambient environment should happen at the moment when the top-layer is just slightly oxidized. Such situation is real since the oxidization process in environment is not fast enough to form a fully-oxidized layer before it is torn up by water. For example, Edmonds et al. [140] established that the oxide growth at the surface of bulk BP declines only after 2 days of exposure in ambient conditions. It is important to note that using phosphorene oxide does not solve the problem of the tunability of 2D BP properties. Wang et al. [133] have found that fundamental properties of phosphorene oxide are also tunable. It was established that the band gap of the phosphorene oxides depends on the degree of phosphorene oxide stoichiometry; an indirect gap is predicted for the non-stoichiometric configurations, whereas a direct gap is predicted for the stoichiometric phosphorene oxide. This means that the use of phosphorene oxide to design humidity sensors does not eliminate the problem of poor stability and irreproducibility of the properties of humidity sensing materials, peculiar to phosphorene.

## 5. Summary

Thus, the analysis performed shows that despite the extraordinary properties of 2D black phosphorus and the high sensitivity of its parameters to humidity, it is premature to expect the appearance of BP-based sensors on the market for humidity sensors. Too many problems associated with improving manufacturing technology and improving the of BP-based sensor parameters must be resolved to ensure that they meet all the requirements for devices designed for the market.

## Figures and Tables

**Table 1:** A compendium of electronic and mechanical properties of phosphorene, graphene and MoS_2_.

Material Type	Phosphorene	Graphene	MoS_2_	h-BN
Conduction type	Semiconductor, ambipolar	Semimetal	Semiconductor, *n*-type	Insulator
Band gap (eV)	0.3–2.0	0	1.2–1.8	5.9
Carrier mobility (cm^2^/V·s)	600–1000	200,000	200	-
On/off ratio	10^3^–10^5^	5.5–44	10^6^–10^8^	-
Thermal conductance (W/m·K)	10–36	2000–5000	34.5–52	250–360
Thermoelectric figure of merit, ZT	0.2–2.2	0	0.4	-
Strain to failure (%)	24–32	19.4–38	19.5–36	24
Young’s modulus (GPa)	35–166	1000	270 ± 100	220–880

*Source:* Reproduced with permission from Ref. [33]. Copyright Elsevier, 2017.

**Table 2:** Methods used for phosphorene preparation.

Process	Precursor	Treatment	Thickness, nm	Ref.
Mechanical exfoliation	BP	Scotch tape on SiO_2_/Si	0.7–6	[73]
ME-PDMS	BP	Scotch tape on SiO_2_/Si, curved PDMS	1.6–2.8	[85]
Hydrothermal	RP, NH_4_F	Teflon lined autoclave, 200 °C	3	[86]
Electrochemical exfoliation	BP, Pt, Na_2_SO_4_	Voltage of +7 V was applied across electrode for 90 min	1.4–10	[87]
Plasma assisted exfoliation	BP	Ar^+^ plasma at 30 W, the pressure of 30 Pa, 20 s	2–10	[82]
LPE	BP, organic solvent/water/ionic liquids	Bath sonication for 24–48 h/tip sonication for 2–4 h, centrifugation at 2000–1000 rpm for 30 min	0.7–6	[73]
CVD	BP thin film over Si (substrate) SnI_4_, Sn	Tube furnace, 950 °C	3.4	[88]
Pulsed layer deposition	BP	KrF (*λ*: 248 nm, *ν*: 5 Hz), 150 °C, vacuum chamber	N/A	[89]

BP—black phosphorous, CVD—chemical vacuum deposition, LPE—liquid phase exfoliation, ME—mechanical exfoliation, PDMS—polydimethylsiloxane, RP—red phosphorous.

**Table 3:** The list of the performances of different humidity sensors based on black phosphorus (BP).

Materials	Transduction Methods	Sensor Response	Response Time (s)	Recovery Time (s)	Test Range (%RH)	Ref.
BP flakes	Resistive	∼4 orders from 10%RH to 85%RH	<1	~1–2	10–85	[95]
BP flakes	Resistive	521% at 97%RH	101	26	11–97	[87]
BP flakes	Resistive	99.17% at 97.3%RH	255	10	11–97	[116]
BP quantum dots	Resistive	∼4 orders from 10%RH to 90%RH	-	-	10–90	[117]
BP/graphene	Resistive	43.4% at 70%RH	9	30	15–70	[118]
BP flakes	Substrate-integrated waveguide (SIW) resonator	197.67 kHz/%RH	-	-	11–97	[119]
		5.82 MHz/%RH at RH >84%	-	-	11–97	[120]
BP flakes	Quartz crystal microbalance (QCM)	82.7 Hz/pg at 90%RH	14	10	10–90	[115,121]
BP flakes	Capacitive	3–4 orders from 11%RH to 97%RH (∆C/C)	4.7	3	11–97	[122]

*Source:* Reproduced with permission from Ref. [32]. Copyright Elsevier, 2018.

## References

[1] Davis R.E., McGregor G.R., Enfield K.B. (2016). Humidity: A review and primer on atmospheric moisture and human health. Environ. Res..

[2] Korotcenkov G. (2018). Why do we need to control humidity?. Handbook of Humidity Measurement: Methods, Materials and Technologies. Vol. 1: Spectroscopic Methods of Humidity Measurement.

[3] The Rotronic Humidity Handbook. http://docplayer.net/13211741-The-rotronic-humidity-handbook.html.

[4] Grange R.I., Hand D.W. (1987). A review of the effects of atmospheric humidity on the growth of horticultural crops. J. Hortic. Sci..

[5] Korotcenkov G. (2018). Handbook of Humidity Measurement: Methods, Materials and Technologies. Vol. 1: Spectroscopic Methods of Humidity Measurement.

[6] Traversa E. (1995). Ceramic sensors for humidity detection: The state–of–the–art and future developments. Sens. Actuators B.

[7] Chen Z., Lu C. (2005). Humidity sensors: A review of materials and mechanisms. Sens. Lett..

[8] Srivastava R. (2012). Humidity sensor: An overview. Int. J. Green Nanotechnol..

[9] Alwis L., Sun T., Grattan K.T.V. (2013). Optical fibre-based sensor technology for humidity and moisture measurement: Review of recent progress. Measurement.

[10] Farahani H., Wagiran R., Hamidon M.N. (2014). Humidity sensors principle; mechanism; and fabrication technologies: A comprehensive review. Sensors.

[11] Blank T.A., Eksperiandova L.P., Beliko K.N. (2016). Recent trends of ceramic humidity sensors development: A review. Sens. Actuators B.

[12] Korotcenkov G. (2019). Handbook of Humidity Measurement: Methods; Materials and Technologies. Vol. 2: Electronic and Electrical Humidity Sensors.

[13] Korotcenkov G. (2020). Handbook of Humidity Measurement: Methods, Materials and Technologies. Vol. 3: Sensing Materials and Technologies.

[14] Fletcher G.F., Galambos J.T. (1963). Phosphorus poisoning in humans. Arch. Int. Med..

[15] Island J.O., Steele G.A., van der Zant H.S., Castellanos-Gomez A. (2015). Environmental instability of few-layer black phosphorus. 2D Mater..

[16] Park C.M., Sohn H.J. (2007). Black phosphorus and its composite for lithium rechargeable batteries. Adv. Mater..

[17] Li L., Yu Y., Ye G.J., Ge Q.Q., Ou X.D., Wu H., Feng D., Chen X.H., Zhang Y. (2014). Black phosphorus field-effect transistors. Nat. Nanotechnol..

[18] Sa B., Li Y.-L., Qi J., Ahuja R., Sun Z. (2014). Strain engineering for phosphorene: The potential application as a photocatalyst. J. Phys. Chem. C.

[19] Xia F., Wang H., Jia Y. (2014). Rediscovering black phosphorus as an anisotropic layered material for optoelectronics and electronics. Nat. Commun..

[20] Abbas A.N., Liu B., Chen L., Ma Y., Cong S., Aroonyadet N., Kopf M., Nilges T., Zhou C. (2015). Black phosphorus gas sensors. ACS Nano.

[21] Shen Z., Sun S., Wang W., Liu J., Liu Z., Jimmy C.Y. (2015). A black-red phosphorus heterostructure for efficient visible-light-driven photocatalysis. J. Mater. Chem. A.

[22] Zhou L., Zhang J., Zhuo Z., Kou L., Ma W., Shao B., Du A., Meng S., Frauenheim T. (2016). Novel excitonic solar cells in phosphorrene-TiO_2_ heterostructures with extraordinary charge separation efficiency. J. Phys. Chem. Lett..

[23] Lee T.H., Kim S.Y., Jang H.W. (2016). Black phosphorus: Critical review and potential for water splitting photocatalyst. Nanomaterials.

[24] Gusmão R., Sofer Z., Pumera M. (2017). Black phosphorus rediscovered: From bulk material to monolayers. Angew. Chem. Int. Ed. Engl..

[25] Jain R., Narayan R., Sasikala S.P., Lee K.E., Jung H.J., Kim S.O. (2017). Phosphorene for energy and catalytic application—Filling the gap between graphene and 2D metal chalcogenides. 2D Mater..

[26] Lin S., Chui Y., Li Y., Lau S.P. (2017). Liquid-phase exfoliation of black phosphorus and its applications. FlatChem.

[27] Yi Y., Yu X.-F., Zhou W., Wang J., Chu P.K. (2017). Two-dimensional black phosphorus: Synthesis; modification; properties; and applications. Mater. Sci. Eng. R.

[28] Choi J.R., Yong K.W., Choi J.Y., Nilgha A., Lin Y., Xu J., Lu X. (2018). Black phosphorus and its biomedical applications. Theranostics.

[29] Irshad R., Tahir K., Li B., Sher Z., Ali J., Nazir S. (2018). A revival of 2D materials; phosphorene: Its application as sensors. J. Ind. Eng. Chem..

[30] Wang W., Xie G., Luo J. (2018). Black phosphorus as a new lubricant. Friction.

[31] Wu S., Hui K.S., Hui K.N. (2018). 2D black phosphorus: From preparation to applications for electrochemical energy storage. Adv. Sci..

[32] Yang A., Wang D., Wang X., Zhang D., Koratkar N., Rong M. (2018). Recent advances in phosphorene as a sensing material. Nano Today.

[33] Khandelwal A., Mani K., Karigerasi M.H., Lahiri I. (2017). Phosphorene—The two-dimensional black phosphorous: Properties; synthesis and applications. Mater. Sci. Eng. B.

[34] Du Y., Ouyang C., Shi S., Lei M. (2010). Ab initio studies on atomic and electronic structures of black phosphorus. J. Appl. Phys..

[35] Wei Q., Peng X. (2014). Superior mechanical flexibility of phosphorene and few-layer black phosphorus. Appl. Phys. Lett..

[36] Appalakondaiah S., Vaitheeswaran G., Lebegue S., Christensen N.E., Svane A. (2012). Effect of van der Waals interactions on the structural and elastic properties of black phosphorus. Phys. Rev. B.

[37] Aldave S.H., Yogeesh M.N., Zhu W.N., Kim J., Sonde S.S., Nayak A.P., Akinwande D. (2016). Characterization and sonochemical synthesis of black phosphorus from red phosphorus. 2D Mater..

[38] Zhang C., Lian J., Yi W., Jiang Y., Liu L., Hu H., Xiao W.D., Du S.X., Sun L.L., Gao H.J. (2009). Surface structures of black phosphorus investigated with scanning tunneling microscopy. J. Phys. Chem. C.

[39] Sun J., Zheng G.Y., Lee H.W., Liu N., Wang H.T., Yao H.B., Yang W., Cui Y. (2014). Formation of stable phosphorus–carbon bond for enhanced performance in black phosphorus nanoparticle–graphite composite battery anodes. Nano Lett..

[40] Liu H., Du Y.C., Deng Y.X., Ye P.D. (2015). Semiconducting black phosphorus: Synthesis; transport properties and electronic applications. Chem. Soc. Rev..

[41] Iwasaki H., Kikegawa T., Fujimura T., Endo S., Akahama Y., Akai T., Shimomura O., Yamaoka S., Yagi T., Akimoto S. (1986). Synchrotron radiation diffraction study of phase transitions in phosphorus at high pressures and temperatures. Phys. B + C.

[42] Ahuja R. (2003). Calculated high pressure crystal structure transformations for phosphorus. Phys. Status Solidi B.

[43] Morita A. (1986). Semiconducting black phosphorus. Appl. Phys. A Mater. Sci. Process..

[44] Takao Y., Morita A. (1981). Electronic structure of black phosphorus: Tight binding approach. Phys. B + C.

[45] Asahina H., Morita A. (1984). Band structure and optical properties of black phosphorus. J. Phys. C Solid State Phys..

[46] Takahashi T., Tokailin H., Suzuki S., Sagawa T., Shirotani I. (1985). Electronic band structure of black phosphorus studied by angle-resolved ultraviolet photoelectron spectroscopy. J. Phys. C: Solid State Phys..

[47] Han C., Yao M., Bai X., Miao L., Zhu F., Guan D., Wang S., Gao C.L., Liu C., Qian D. (2014). Electronic structure of black phosphorus studied by angle-resolved photoemission spectroscopy. Phys. Rev. B.

[48] Rudenko A.N., Katsnelson M.I. (2014). Quasiparticle band structure and tight-binding model for single-and bilayer black phosphorus. Phys. Rev. B.

[49] Wu R.J., Topsakal M., Low T., Robbins M.C., Haratipour N., Jeong J.S., Wang S., Gao C.L., Liu C., Qian D. (2015). Atomic and electronic structure of exfoliated black phosphorus. J. Vac. Sci. Technol. A.

[50] Xu Y., Dai J., Zeng X.C. (2015). Electron-transport properties of few-layer black phosphorus. J. Phys. Chem. Lett..

[51] Carvalho A., Wang M., Zhu X., Rodin A.S., Su H., Neto A.H.C. (2016). Phosphorene: From theory to applications. Nat. Rev. Mater..

[52] Rahman M.Z., Kwong C.W., Davey K., Qiao S.Z. (2016). 2D phosphorene as a water splitting photocatalyst: Fundamentals to applications. Energy Environ. Sci..

[53] Cai Y., Zhang G., Zhang Y.-W. (2014). Layer-dependent band alignment and work function of few-layer phosphorene. Sci. Rep..

[54] Liu H., Neal A.T., Zhu Z., Luo Z., Xu X.F., Tománek D., Ye P.D. (2014). Phosphorene: An unexplored 2D semiconductor with a high hole mobility. ACS Nano.

[55] Wang Q.H., Kalantar-Zadeh K., Kis A., Coleman J.N., Strano M.S. (2012). Electronics and optoelectronics of two-dimensional transition metal dichalcogenides. Nat. Nanotechnol..

[56] Lv R.T., Robinson J.A., Schaak R.E., Sun D., Sun Y.F., Mallouk T.E., Terrones M. (2015). Transition metal dichalcogenides and beyond: Synthesis; properties; and applications of single and few-layer nanosheets. Acc. Chem. Res..

[57] Yang J., Xu R., Pei J., Myint Y.W., Wang F., Wang Z., Zhang S., Yu Z., Lu Y. (2014). Unambiguous identification of monolayer phosphorene by phase-shifting interferometry. arXiv.

[58] Zhang S., Yang J., Xu R., Wang F., Li W., Ghufran M., Zhang Y.-W., Yu Z., Zhang G., Qin Q. (2014). Extraordinary photoluminescence and strong temperature/angle-dependent Raman responses in few-layer phosphorene. ACS Nano.

[59] Hanlon D., Backes C., Doherty E., Cucinotta C.S., Berner N.C., Boland C., Lee K., Harvey A., Lynch P., Gholamyand Z. (2015). Liquid exfoliation of solvent-stabilised black phosphorus: Applications beyond electronics. Nat. Commun..

[60] Qiao J., Kong X., Hu Z.-X., Yang F., Ji W. (2014). High-mobility transport anisotropy and linear dichroism in few-layer black phosphorus. Nat. Commun..

[61] Koenig S.P., Doganov R.A., Seixas L., Carvalho A., Tan J.Y., Taniguchi T., Yakovlev N., Castro Neto A.H., Ozyilmaz B. (2016). Electron doping of ultrathin black phosphorus with Cu adatoms. Nano Lett..

[62] Yu X., Zhang S., Zeng H., Wang Q.J. (2016). Lateral black phosphorene p-n junctions formed via chemical doping for high performance near-infrared photodetector. Nano Energy.

[63] Das S., Demarteau M., Roelofs A. (2014). Ambipolar phosphorene field effect transistor. ACS Nano.

[64] Schwierz F. (2010). Graphene transistors. Nat. Nanotechnol..

[65] Sevik C., Sevinçli H. (2016). Promising thermoelectric properties of phosphorenes. Nanotechnology.

[66] Zhou H., Cai Y., Zhang G., Zhang Y.-W. (2016). Thermoelectric properties of phosphorene at the nanoscale. J. Mater. Sci..

[67] Dai J., Zeng X.C. (2014). Bilayer phosphorene: Effect of stacking order on bandgap and its potential applications in thin-film solar cells. J. Phys. Chem. Lett..

[68] Ray S.R. (2016). First-principles study of MoS_2_, phosphorene and graphene based single electron transistor for gas sensing applications. Sens. Actuators B.

[69] Donarelli M., Ottaviano L. (2018). 2D materials for gas sensing applications: A review on graphene oxide, MoS_2_, WS_2_ and phosphorene. Sensors.

[70] Lv H.Y., Lu W.J., Shao D.F., Sun Y.P. (2014). Enhanced thermoelectric performance of phosphorene by strain-induced band convergence. Phys. Rev. B.

[71] Li X.-B., Guo P., Cao T.-F., Liu H., Lau W.-M., Liu L.-M. (2016). Structures, stabilities, and electronic properties of defects in monolayer black phosphorus. Sci. Rep..

[72] Akhtar M., Anderson G., Zhao R., Alruqi A., Mroczkowska J.E., Sumanasekera G., Jasinski J.B. (2017). Recent advances in synthesis; properties; and applications of phosphorene. NpJ 2D Mater. Appl..

[73] Dhanabalan S.C., Ponraj J.S., Guo Z., Li S., Bao Q., Zhang H. (2017). Emerging trends in phosphorene fabrication towards next generation devices. Adv. Sci..

[74] Bridgman P.W. (1914). Two new modifications of phosphorus. J. Am. Chem. Soc..

[75] Maruyama Y., Suzuki S., Kobayashi K., Tanuma S. (1981). Synthesis and some properties of black phosphorus single crystals. Phys. B + C.

[76] Shirotani I. (1982). Growth of large single crystals of black phosphorus at high pressures and temperatures; and its electrical properties. Mol. Cryst. Liq. Cryst..

[77] Endo S., Akahama Y., Terada S., Narita S. (1982). Growth of large single crystals of black phosphorus under high pressure. Jpn. J. Appl. Phys..

[78] Krebs H., Schultze-Gebhardt F. (1955). Über die Struktur und Eigenschaften der Halbmetalle. VII. Neubestimmung der Struktur des glasigen Selens nach verbesserten röntgenographischen Methoden. Acta Crystallogr..

[79] Lange S., Schmidt P., Nilges T. (2007). Au_3_SnP_7_@black phosphorus: An easy access to black phosphorus. Inorg. Chem..

[80] Nilges T., Kersting M., Pfeifer T. (2008). A fast low-pressure transport route to large black phosphorus single crystals. J. Solid State Chem..

[81] Köpf M., Eckstein N., Pfister D., Grotz C., Krüger I., Greiwe M., Hansen T., Kohlmann H., Nilges T. (2014). Access and in situ growth of phosphorene-precursor black phosphorus. J. Cryst. Growth.

[82] Lu W.L., Nan H.Y., Hong J.H., Chen Y.M., Zhu C., Liang Z., Ma X., Ni Z., Jin C., Zhang Z. (2014). Plasma-assisted fabrication of monolayer phosphorene and its Raman characterization. Nano Res..

[83] Jia J., Jang S.K., Lai S., Xu J., Choi Y.J., Park J.H., Lee S. (2015). Plasma-treated thickness-controlled two-dimensional black phosphorus and its electronic transport properties. ACS Nano.

[84] Lee G., Lee J.Y., Lee G.H., Kim J. (2016). Tuning the thickness of black phosphorus via ion bombardment-free plasma etching for device performance improvement. J. Mater. Chem. C.

[85] Andres C.-G., Vicarelli L., Prada E., Island J.O., Narasimha-Acharya K.L., Blanter S.I., Groenendijk D.J., Buscema M., Steele G.A., Alvarez J.V. (2014). Isolation and characterization of fewlayer black phosphorus. 2D Mater..

[86] Zhao G., Wang T., Shao Y., Wu Y., Huang B., Hao X. (2017). A novel mild phase-transition to prepare black phosphorus nanosheets with excellent energy applications. Small.

[87] Erande M.B., Pawar M.S., Late D.J. (2016). Humidity sensing and photodetection behavior of electrochemically exfoliated atomically thin-layered black phosphorus nanosheets. ACS Appl. Mater. Interfaces.

[88] Joshua B.S., Daniel H., Hai-Feng J. (2016). Growth of 2D black phosphorus film from chemical vapor deposition. Nanotechnology.

[89] Yang Z., Hao J., Yuan S., Lin S., Yau H.M., Dai J., Lau S.P. (2015). Field-effect transistors based on amorphous black phosphorus ultrathin films by pulsed laser deposition. Adv. Mater..

[90] Cui S., Pu H., Wells S.A., Wen Z., Mao S., Chang J., Hersam M.C., Chen J. (2015). Ultrahigh sensitivity and layer-dependent sensing performance of phosphorene-based gas sensors. Nat. Commun..

[91] Huo C., Yan Z., Song X., Zeng H. (2015). 2D materials via liquid exfoliation: A review on fabrication and applications. Sci. Bull..

[92] Brent J.R., Savjani N., Lewis E.A., Haigh S.J., Lewis D.J., O’Brien P. (2014). Production of fewlayer phosphorene by liquid exfoliation of black phosphorus. Chem. Commun..

[93] Guo Z., Zhang H., Lu S., Wang Z., Tang S., Sha J., Sun Z., Xie H., Wang H., Yu X.-F. (2015). From black phosphorus to phosphorene: Basic solvent exfoliation; evolution of Raman scattering; and applications to ultrafast photonics. Adv. Funct. Mater..

[94] Yasaei P., Kumar B., Foroozan T., Wang C., Asadi M., Tuschel D., Indacochea J.E., Klie R.F., Salehi-Khojin A. (2015). High-quality black phosphorus atomic layers by liquid-phase exfoliation. Adv. Mater..

[95] Yasaei P., Behranginia A., Foroozan T., Asadi M., Kim K., Khalili-Araghi F., Salehi-Khojin A. (2015). Stable and selective humidity sensing using stacked black phosphorus flakes. ACS Nano.

[96] Lewis E.A., Brent J.R., Derby B., Haigh S.J., Lewis D.J. (2017). Solution processing of two-dimensional black phosphorus. Chem. Commun..

[97] Nicolosi V., Chhowalla M., Kanatzidis M.G., Strano M.S., Coleman J.N. (2013). Liquid exfoliation of layered materials. Science.

[98] Ang P.K., Wang S., Bao Q., Thong J.T.L., Loh K.P. (2009). High-throughput synthesis of graphene by intercalation-exfoliation of graphite oxide and study of ionic screening in graphene transistor. ACS Nano.

[99] Zeng X.M., Yan H.J., Ouyang C.Y. (2012). First principles investigation of dynamic performance in the process of lithium intercalation into black phosphorus. Acta Phys. Sin. Chin. Ed..

[100] Sun J., Lee H.-W., Pasta M., Yuan H., Zheng G., Sun Y., Li Y., Cui Y. (2015). A phosphorene–graphene hybrid material as a high-capacity anode for sodium-ion batteries. Nat. Nanotechnol..

[101] Kang J., Wood J.D., Wells S.A., Lee J.-H., Liu X., Chen K.-S., Hersam M.C. (2015). Solvent exfoliation of electronic-grade, two-dimensional black phosphorus. ACS Nano.

[102] Wood J.D., Wells S.A., Jariwala D., Chen K.-S., Cho E., Sangwan V.K., Liu X., Lauhon L.J., Marks T.J., Hersam M.C. (2014). Effective passivation of exfoliated black phosphorus transistors against ambient degradation. Nano Lett..

[103] Kang J., Wells S.A., Wood J.D., Lee J.-H., Liu X., Ryder C.R., Zhu J., Guest J.R., Husko C.A., Hersam M.C. (2016). Stable aqueous dispersions of optically and electronically active phosphorene. Proc. Natl. Acad. Sci. USA.

[104] Xu J.-Y., Gao L.-F., Hu C.-X., Zhu Z.-Y., Zhao M., Wang Q., Zhang H.-L. (2016). Preparation of large size, few-layer black phosphorus nanosheets via phytic acid-assisted liquid exfoliation. Chem. Commun..

[105] Kumar V., Brent J.R., Shorie M., Kaur H., Chadha G., Thomas A.G., Lewis E.A., Rooney A., Nguyen L., Zhong X.L. (2016). Nanostructured aptamer-functionalized black phosphorus sensing platform for label-free detection of myoglobin, a cardiovascular disease biomarker. ACS Appl. Mater. Interfaces.

[106] Brent J.R., Ganguli A.K., Kumar V., Lewis D.J., McNaughter P.D., O’Brien P., Sabherwal P., Tedstone A.A. (2016). On the stability of surfactant-stabilised few-layer black phosphorus in aqueous media. RSC Adv..

[107] Wang H., Yang X., Shao W., Chen S., Xie J., Zhang X., Wang J., Xie Y. (2015). Ultrathin black phosphorus nanosheets for efficient singlet oxygen generation. J. Am. Chem. Soc..

[108] Erande M.B., Suryawanshi S.R., More M.A., Late D.J. (2015). Electrochemically exfoliated black phosphorus nanosheets—Prospective field emitters. Eur. J. Inorg. Chem..

[109] Mayorga-Martinez C.C., Latiff N.M., Eng A.Y.S., Sofer Z., Pumera M. (2016). Black phosphorus nanoparticle labels for immunoassays via hydrogen evolution reaction mediation. Anal. Chem..

[110] Yoo D., Kim M., Jeong S., Han J., Cheon J. (2014). Chemical synthetic strategy for single-layer transition-metal chalcogenides. J. Am. Chem. Soc..

[111] Zhang Y., Rui X., Tang Y., Liu Y., Wei J., Chen S., Li W., Liu Y., Deng J., Ma B. (2016). Wet-chemical processing of phosphorus composite nanosheets for high-rate and high-capacity Lithium-ion batteries. Adv. Energy Mater..

[112] Bagher S., Mansouri N., Aghaie E. (2016). Phosphorene: A new competitor for graphene. Int. J. Hydrogen Energy.

[113] Chen X., Dobson J.F., Raston C.L. (2012). Vortex fluidic exfoliation of graphite and boron nitride. Chem. Commun..

[114] Xu F., Ge B., Chen J., Nathan A., Xin L.L., Ma H., Min H., Zhu C., Xia W., Li Z. (2016). Scalable shear-exfoliation of high-quality phosphorene nanoflakes with reliable electrochemical cycleability in nano batteries. 2D Mater..

[115] Yao Y., Zhang H., Sun J., Ma W., Li L., Li W., Du J. (2017). Novel QCM humidity sensors using stacked black phosphorus nanosheets as sensing film. Sens. Actuators B.

[116] Late D.J. (2016). Liquid exfoliation of black phosphorus nanosheets and its application as humidity sensor. Microporous Mesoporous Mater..

[117] Zhu C., Feng X., Zhang L., Li M., Chen J., Xu S., Huang G., Chen W., Sun L. (2016). Ultrafast preparation of black phosphorus quantum dots for efficient humidity sensing. Chem. Eur. J..

[118] Phan D.-T., Park I., Park A.-R., Park C.-M., Jeon K.-J. (2017). Black P/graphene hybrid: A fast response humidity sensor with good reversibility and stability. Sci. Rep..

[119] Chen C.M., Xu J., Yao Y. (2017). SIW resonator humidity sensor based on layered black phosphorus. Electron. Lett..

[120] Chen C.-M., Xu J. (2018). A miniaturized evanescent mode HMSIW humidity sensor. Int. J. Microw. Wirel. Technol..

[121] Walia S., Sabri Y., Ahmed T., Field M.R., Ramanathan R., Arash A., Bhargava S.K., Sriram S., Bhaskaran M., Bansal V. (2017). Defining the role of humidity in the ambient degradation of few-layer black phosphorus. 2D Mater..

[122] He P., Brent J.R., Ding H., Yang J., Lewis D.J., Brien P.O., Derby B. (2018). Fully printed high performance humidity sensors based on two-dimensional materials. Nanoscale.

[123] Lee G., Kim S., Jung S., Jang S., Kim J. (2017). Suspended black phosphorus nanosheet gas sensors. Sens. Actuators B.

[124] Donarelli M., Ottaviano L., Giancaterini L., Fioravanti G., Perrozzi F., Cantalini C. (2016). Exfoliated black phosphorus gas sensing properties at room temperature. 2D Mater..

[125] Miao J., Cai L., Zhang S., Nah J., Yeom J., Wang C. (2017). Air-stable humidity sensor using few-layer black phosphorus. ACS Appl. Mater. Interfaces.

[126] Cai Y.Q., Zhang G., Zhang Y.W. (2015). Electronic properties of phosphorene/graphene and phosphorene/hexagonal boron nitride heterostructures. J. Phys. Chem. C.

[127] Das S., Zhang W., Demarteau M., Hoffmann A., Dubey M., Roelofs A. (2014). Tunable transport gap in phosphorene. Nano Lett..

[128] Kim J., Baik S.S., Ryu S.H., Sohn Y., Park S., Park B.-G., Denlinger J., Yi Y., Choi H.J., Kim K.S. (2015). Observation of tunable band gap and anisotropic dirac semimetal state in black phosphorus. Science.

[129] Tran V., Soklaski R., Liang Y., Yang L. (2014). Layer-controlled band gap and anisotropic excitons in few-layer black phosphorus. Phys. Rev. B.

[130] Conley H.J., Wang B., Ziegler J.I., Haglund R.F., Pantelides S.T., Bolotin K.I. (2013). Bandgap engineering of strained monolayer and bilayer MoS_2_. Nano Lett..

[131] Zhou Q., Chen Q., Tong Y., Wang J. (2016). Light-induced ambient degradation of few-layer black phosphorus: Mechanism and protection. Angew. Chem. Int. Ed..

[132] Rodin A.S., Carvalho A., Castro Neto A.H. (2014). Strain-induced gap modification in black phosphorus. Phys. Rev. Let..

[133] Wang G., Pandey R., Karna S.P. (2015). Phosphorene oxide: Stability and electronic properties of a novel two-dimensional material. Nanoscale.

[134] Ma X., Lu W., Chen B., Zhong D., Huang L., Dong L., Jin C., Zhang Z. (2015). Performance change of few layer black phosphorus transistors in ambient. AIP Adv..

[135] Ling X., Wang H., Huang S.X., Xia F.N., Dresselhaus M.S. (2015). The renaissance of black phosphorus. Proc. Natl. Acad. Sci. USA.

[136] Favron A., Gaufrès E., Fossard F., Phaneuf-L’Heureux A.-L., Tang N.Y., Lévesque P.L., Lioseau A., Leonelli R., Francoeur S., Martel R. (2015). Photooxidation and quantum confinement effects in exfoliated black phosphorus. Nat. Mater..

[137] Huang Y., Qiao J., He K., Bliznakov S., Sutter E., Chen X., Luo D., Meng F., Su D., Decker J. (2016). Degradation of black phosphorus: The role of oxygen and water. Chem. Mater..

[138] Wang G., Slough W.J., Pandey R., Karna S.P. (2016). Degradation of phosphorene in air: Understanding at atomic level. 2D Mater..

[139] Ziletti A., Carvalho A., Campbell D.K., Coker D.F., Neto A.C. (2015). Oxygen defects in phosphorene. Phys. Rev. Lett..

[140] Edmonds M.T., Tadich A., Carvalho A., Ziletti A., Donnell K.M.O., Koenig S.P., Coker D.F., Ozyilmaz B., Castro Neto A.H., Fuhrer M.S. (2015). Creating a stable oxide at the surface of black phosphorus. ACS Appl. Mater. Interfaces.

[141] Liu X., Wood J.D., Chen K.-S., Cho E., Hersam M.C. (2015). In situ thermal decomposition of exfoliated two-dimensional black phosphorus. J. Phys. Chem. Lett..

[142] Li P., Zhang D., Liu J., Chang H., Sun Y., Yin N. (2015). Air-stable black phosphorus devices for ion sensing. ACS Appl. Mater Interfaces.

[143] Koenig S.P., Doganov R.A., Schmidt H., Neto A.H.C., Ozyilmaz B. (2014). Electric field effect in ultrathin black phosphorus. Appl. Phys. Lett..

[144] Wan B.S., Yang B.C., Wang Y., Zhang J.Y., Zeng Z.M., Liu Z.Y., Wang W. (2015). Enhanced stability of black phosphorus field-effect transistors with SiO_2_ passivation. Nanotechnology.

[145] Son Y., Kozawa D., Liu A.T., Koman V.B., Wang Q.H., Strano M.S. (2017). A study of bilayer phosphorene stability under MoS_2_-passivation. 2D Mater..

[146] Xing C., Jing G., Liang X., Qiu M., Li Z., Cao R., Li X., Fan D., Zhang H. (2017). Graphene oxide/black phosphorus nanoflake aerogels with robust thermo-stability and significantly enhanced photothermal properties in air. Nanoscale.

[147] Tan S.J.R., Abdelwahab I., Chu L., Poh S.M., Liu Y., Lu J., Lu J., Chen W., Loh K.P. (2018). Quasi-monolayer black phosphorus with high mobility and air stability. Adv. Mater..

[148] Zhang Y., Dong N., Tao H., Yan C., Huang J., Liu T., Robertson A.W., Texter J., Wang J., Sun Z. (2017). Exfoliation of Stable 2D black phosphorus for device fabrication. Chem. Mater..

[149] Zhang H. (2015). Ultrathin two-dimensional nanomaterials. ACS Nano.

[150] Ziletti A., Carvalho A., Trevisanutto P.E., Campbell D.K., Coker D.F., Castro Neto A.H. (2015). Phosphorene oxides: Bandgap engineering of phosphorene by oxidation. Phys. Rev. B.

